# Supplementary material for: Aromatic nonpolar organogels for efficient and stable perovskite green emitters
Source: Nat Commun. 2020 Sep 15;11:4638. doi: 10.1038/s41467-020-18383-y (PMC7493929; doi:10.1038/s41467-020-18383-y)
Supplement: Supplementary file 1 — Supplementary Information [file 41467_2020_18383_MOESM1_ESM.pdf]

Supplementary Information for

Aromatic nonpolar organogels for efficient and stable perovskite green emitters

Park et al.

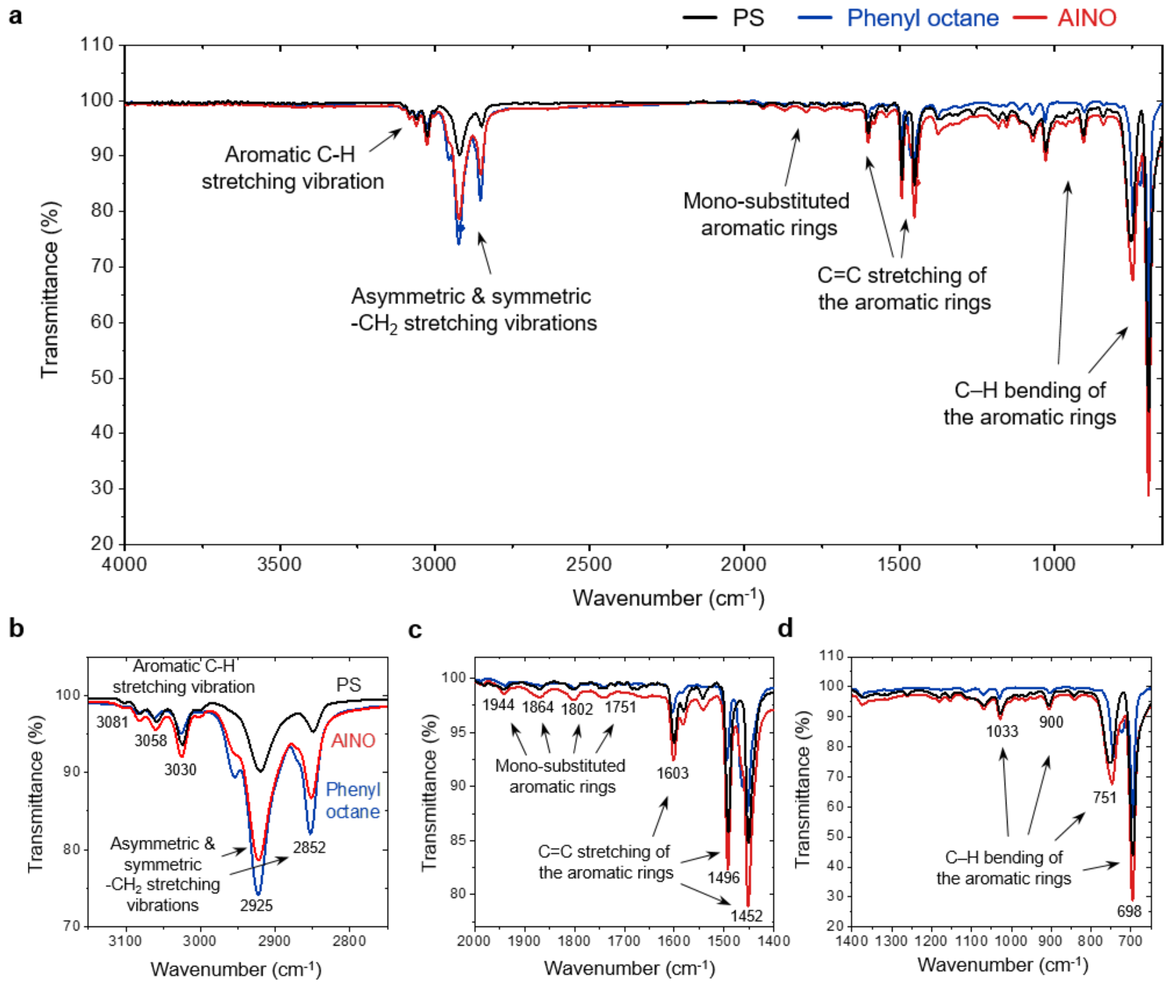

Supplementary Fig. 1. Fourier transform infrared (FT-IR) spectroscopy investigations. a, FT-IR spectra of the AINO and its components: polystyrene (PS) and phenyl octane. **b-d,** Zoom-in FT-IR spectra of the AINO and its components in partial wavenumber range 2,750 to 3,150 cm^{-1} (**b**), 1,400 to 2,000 cm^{-1} (**c**), and 650 to 1,400 cm^{-1} (**d**), respectively.

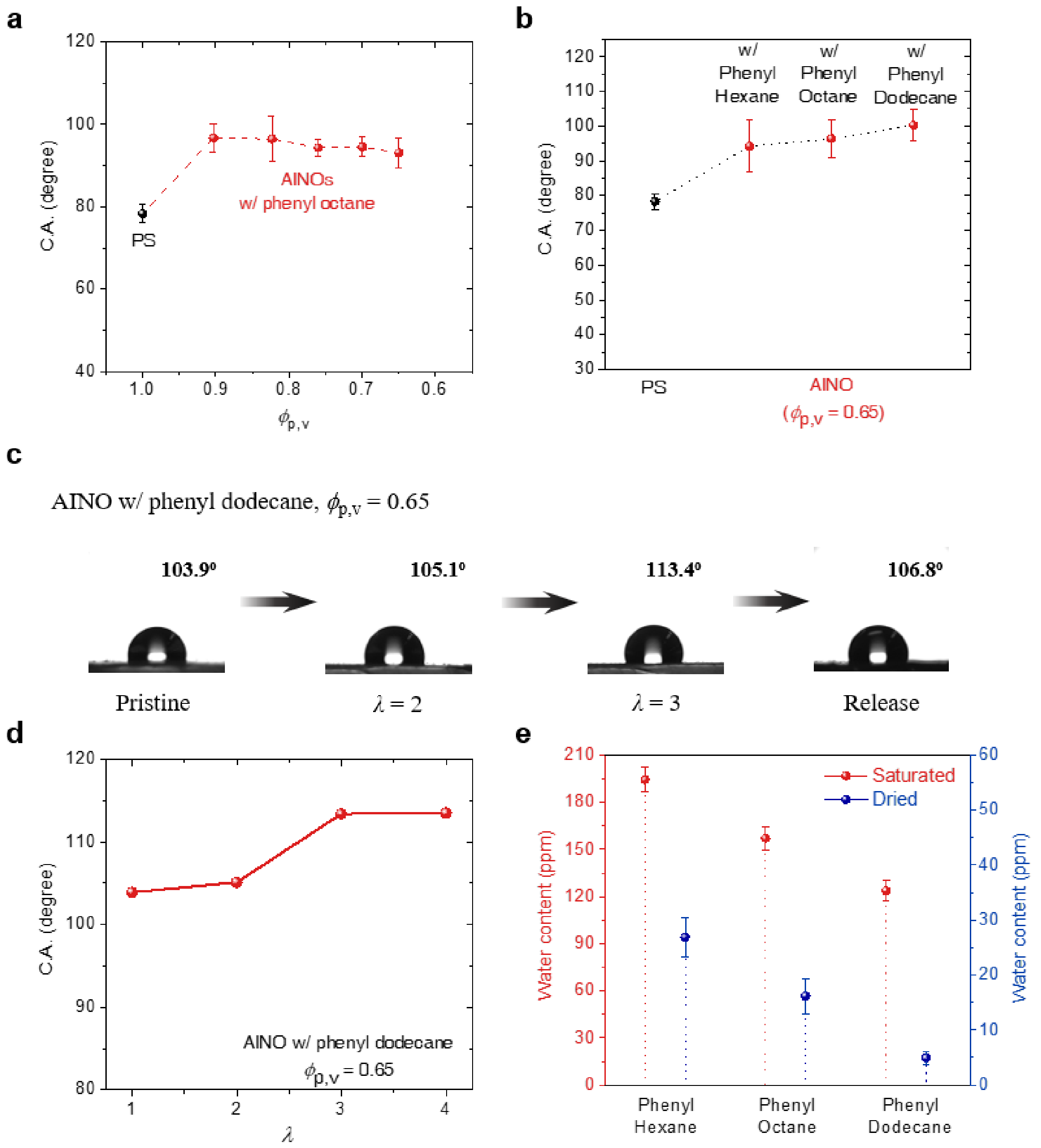

Supplementary Fig. 2. Hydrophobicity of the AINOs. a, Contact angles of deionized water micro-droplets on the AINOs as a function of their polymer volume concentration. **b,** Contact angles of the micro-droplets on the AINOs according to their solvent type. **c,** Photographs of the micro-droplet on the AINO while stretching. **d,** The AINO maintains its hydrophobicity while stretching. **e,** Water solubility of phenyl alkane solvents. The red columns are the solubility of the phenyl alkanes in water saturated state, while the blue columns are the solubility of the phenyl alkanes in dried state. Values in **a**, **b**, and **e** represent the mean and standard deviation (n = 3).

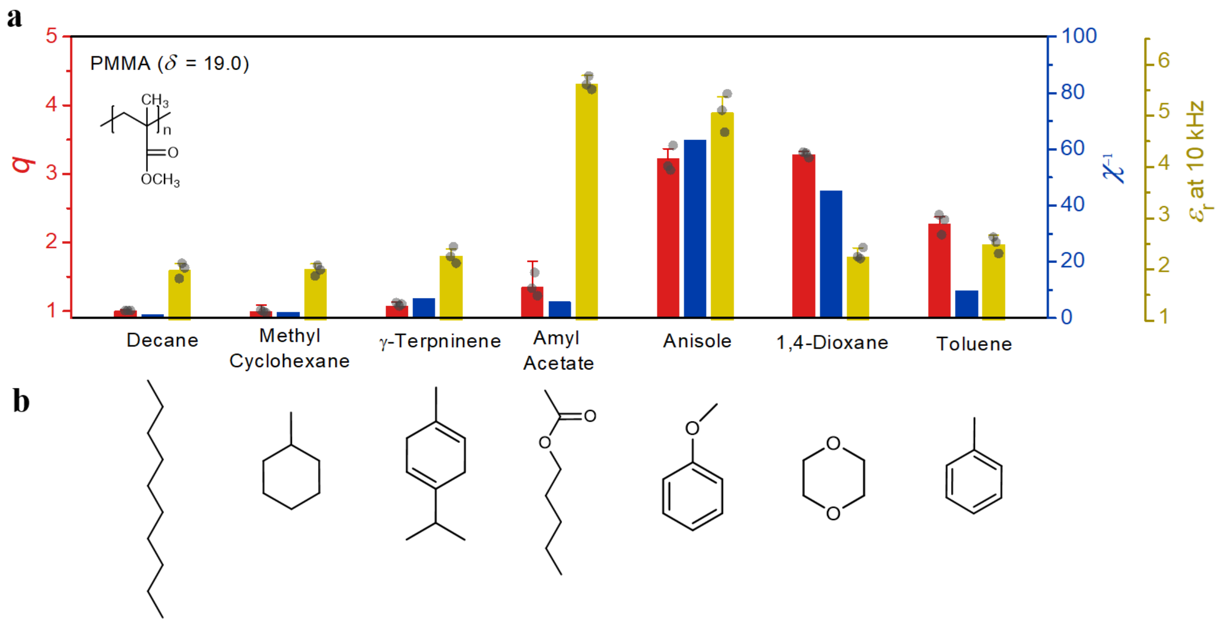

Supplementary Fig. 3. Materials investigations for PMMA-based nonpolar organogels. a, Swelling ratio and interaction affinity for various nonpolar solvents with PMMA, and their static dielectric constant at 10 kHz. Data represent the mean and standard deviation (n = 3). **b,** Molecular structures of the corresponding nonpolar solvents in **a**. Although the polymer and solvents have the higher values of dielectric constant and interaction affinity, their swelling ratios are lower than the combination of aromatic nonpolar polymer and solvents.

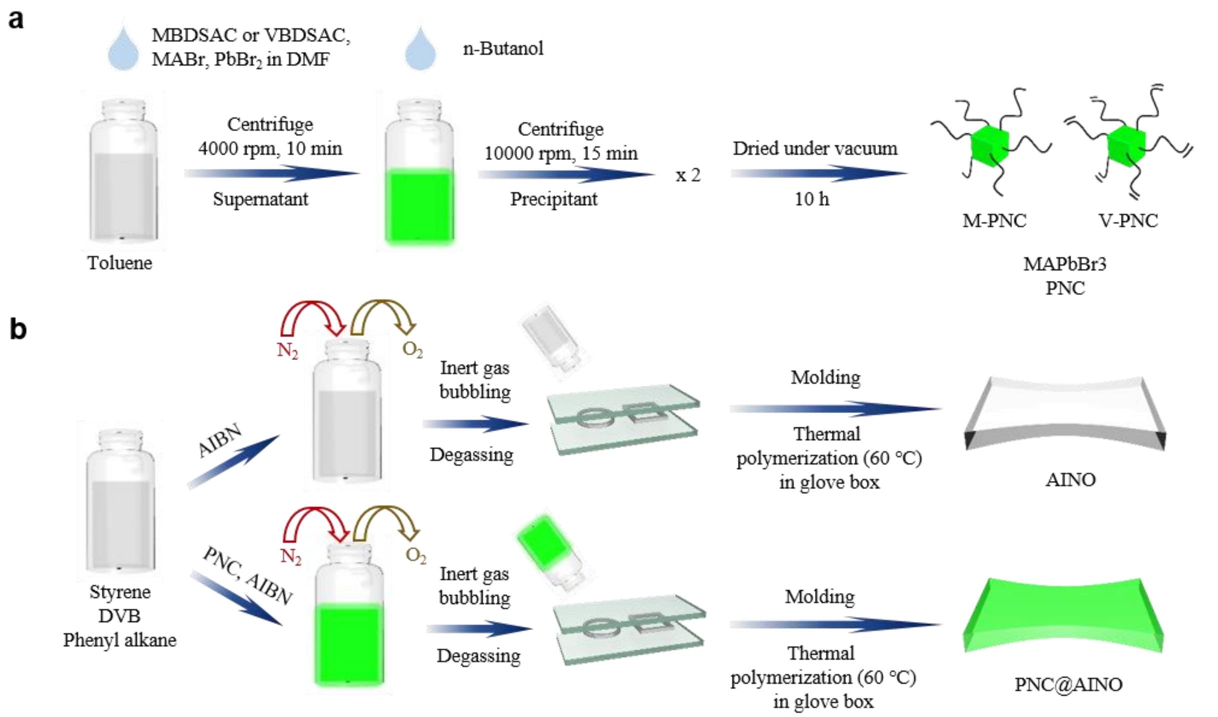

Supplementary Fig. 4. Fabrication procedure of the PNCs, AINOs, and PNC@AINO. a, Schematic illustration of the synthesis procedure of the PNCs. Briefly, the ligands and the PNC precursors dissolved in DMF are added into toluene under stirring, and the supernatants are collected after centrifugation. The supernatants are washed with n-butanol twice, followed by drying under vacuum for 10 hours. MBDSAC, 4-methylbenzylstearylammonium chloride; VBDSAC, 4-vinylbenzyldimethylammonium chloride; MABr, methylammonium bromide; $PbBr_2$, lead bromide; DMF; n,n-dimethylformamide. **b,** Schematic illustration of the synthesis procedure of the AINOs and PNC@AINOs. For the AINO the precursors are dissolved with desired ratio, and degassed by multiple freeze–pump–thaw and inert gas bubbling cycles. The precursor solution is poured into the mold, and thermally polymerized under inert atmosphere. The synthesis procedure of the PNC@AINOs is almost same with that of the AINOs, excepting the addition of the PNCs before degassing. DVB, divinylbenzene; AIBN, 2,2’-azobisisobutyronitrile.

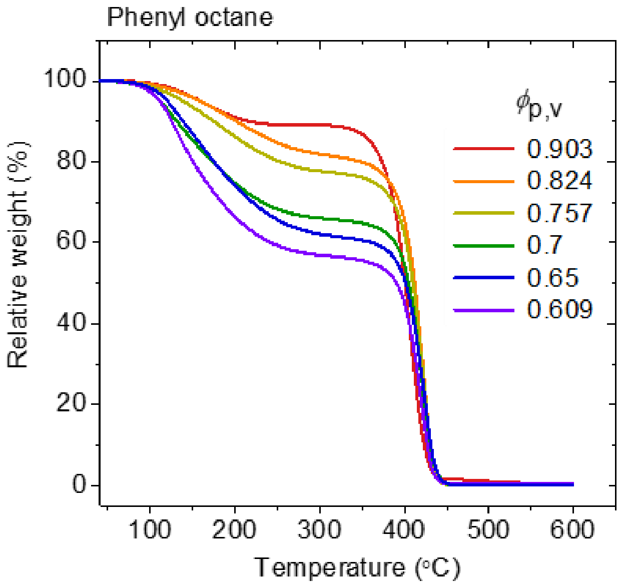

Supplementary Fig. 5. Thermogravimetric analysis (TGA) of the AINOs. The phenyl octane solvent in the AINOs evaporates in range 100 to 250 ℃, and the PS polymer in the AINOs decompose around 400 ℃. The polymer volume concentration of the AINOs is calculated from the relative weights of the solvent and the polymer in the AINOs. The calculation results accord with the initially added volume of the solvent for synthesizing the AINOs.

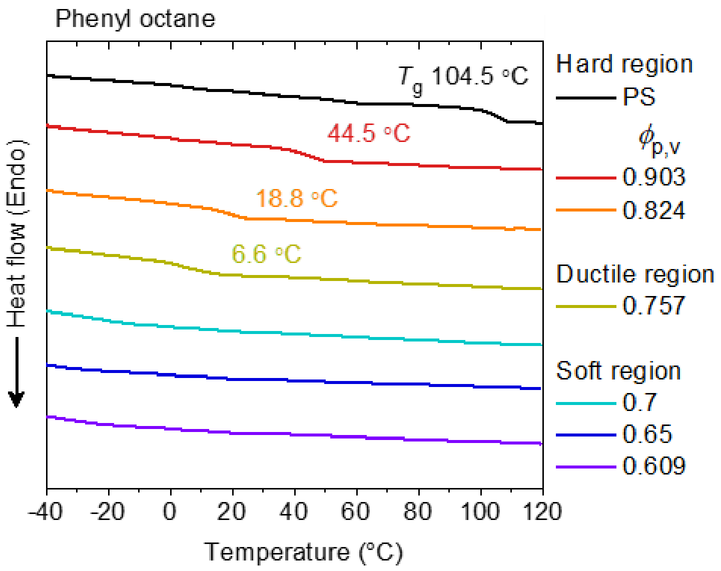

Supplementary Fig. 6. Differential scanning calorimetry (DSC) results of the AINOs. The solvent in the AINOs acts as plasticizers that lowers the glass transition temperature (T_g) of the AINO. The pure PS has T_g of 104.5 °C. The small amount of the solvent lowers its T_g effectively, reaching 6.6 °C for the AINO that has polymer volume concentration ($\phi_{p,v}$) of 0.757. When the $\phi_{p,v}$ of the AINOs is below 0.7, the T_g of the AINOs is not detected in temperature region above -40 °C, which value is near the melting point of phenyl octane.

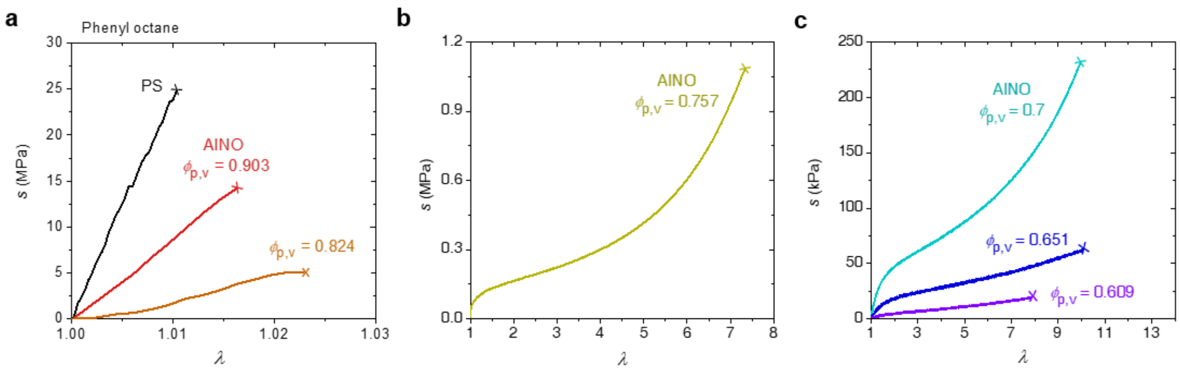

Supplementary Fig. 7. Tensile stress–stretch tests for the AINOs. a, Tensile stress–stretch curves for PS and the AINOs having brittle mechanical behaviors. **b,** Tensile stress–stretch curve for the AINO having ductile elastomeric mechanical behavior. **c,** Tensile stress–stretch curves for the AINOs having soft elastomeric mechanical behaviors.

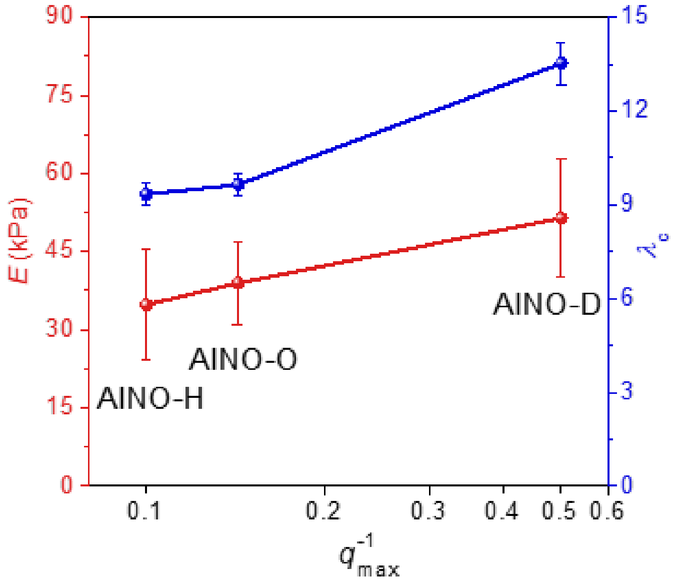

Supplementary Fig. 8. Effect of solvents on the mechanical behaviors of the AINOs. Elastic modulus and rupture stretch of the AINOs varying types of phenyl alkanes at polymer volume concentration of 0.65, as a function of their reciprocal values of the maximum swelling ratio. AINO-H, phenyl hexane based AINO; AINO-O, phenyl octane based AINO; AINO-D, phenyl dodecane based AINO. Values represent the mean and standard deviation (n = 3–5).

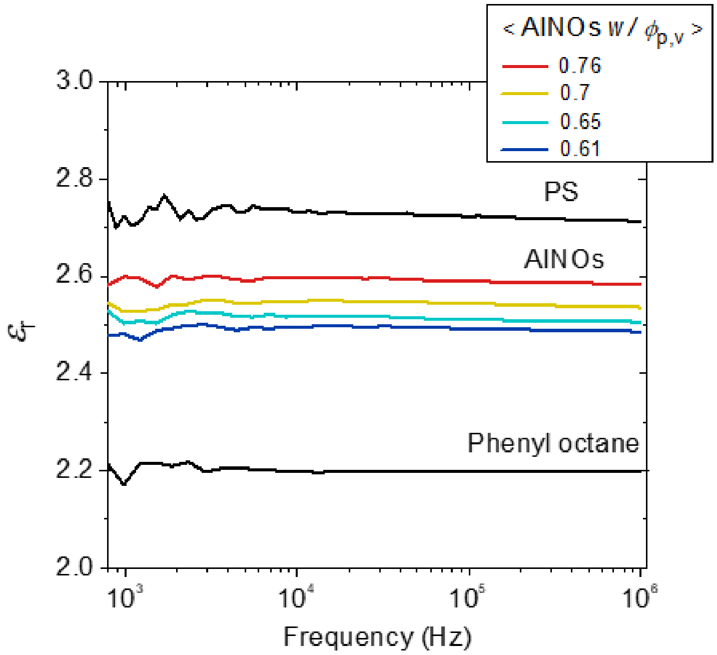

Supplementary Fig. 9. Polarity of the AINOs. The static dielectric constant of the elastomeric AINOs and their parents as a function of frequency.

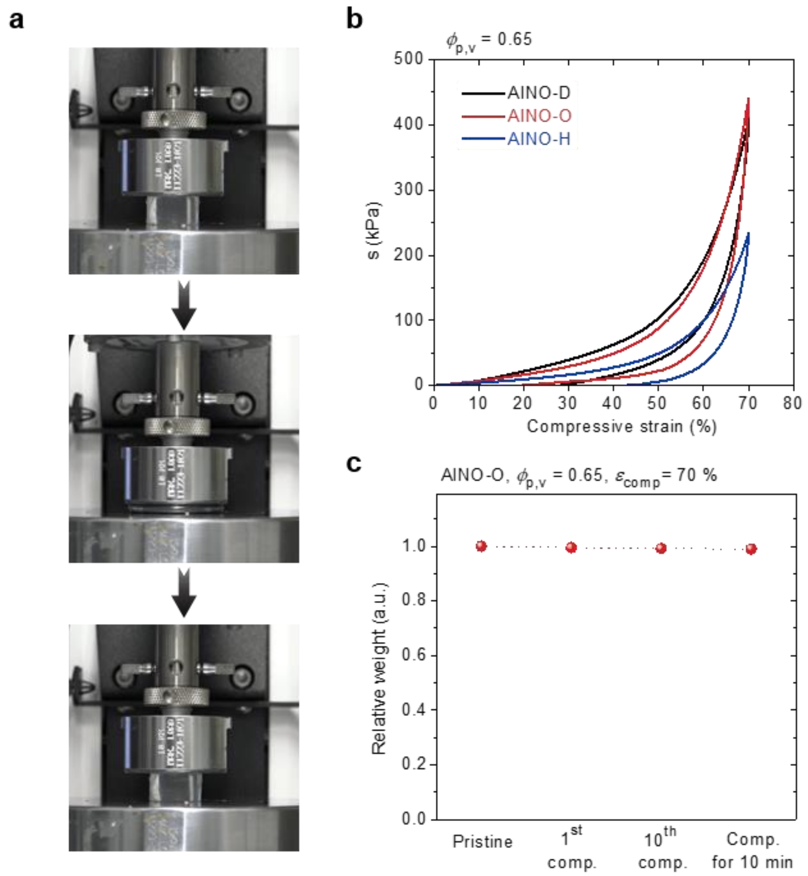

Supplementary Fig. 10. Mechanical compression tests of the AINOs. a, Photographs of the AINO undergoing compression test. Solvent and polymer volume concentration of the AINO are phenyl octane and 0.65, respectively. **b,** Compressive stress–strain curves of the AINOs varying types of phenyl alkanes at polymer volume concentration of 0.65. **c,** The relative weight of the AINOs is maintained from the pristine state to the first compression, followed by the 10th compression, and after compression for 10 min. The compressive strain of tests is fixed to 70 %.

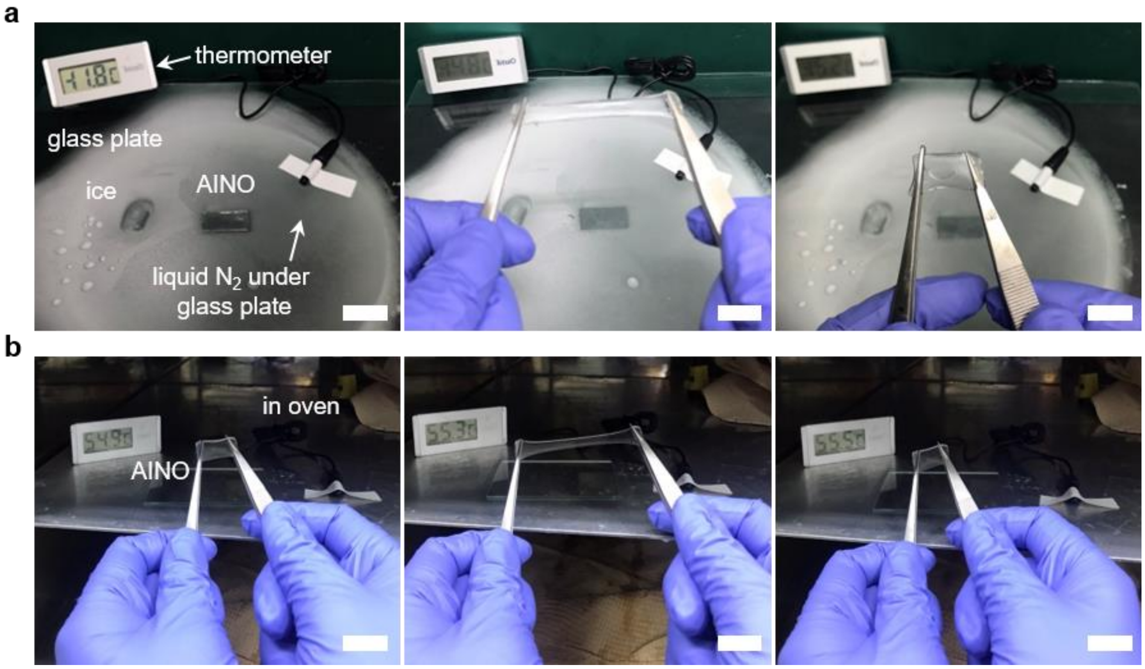

Supplementary Fig. 11. Temperature stability of the AINO. a, Photographs of the AINO at temperature of below -10 ℃. The water droplets freeze on the glass plate, cooled down by liquid nitrogen, while the AINO maintains its transparency, stretchability, and anti-icing property on its surface. **b,** Photographs of the AINO stretched in oven at temperature of above 50 ℃. Scale bar = 2 cm.

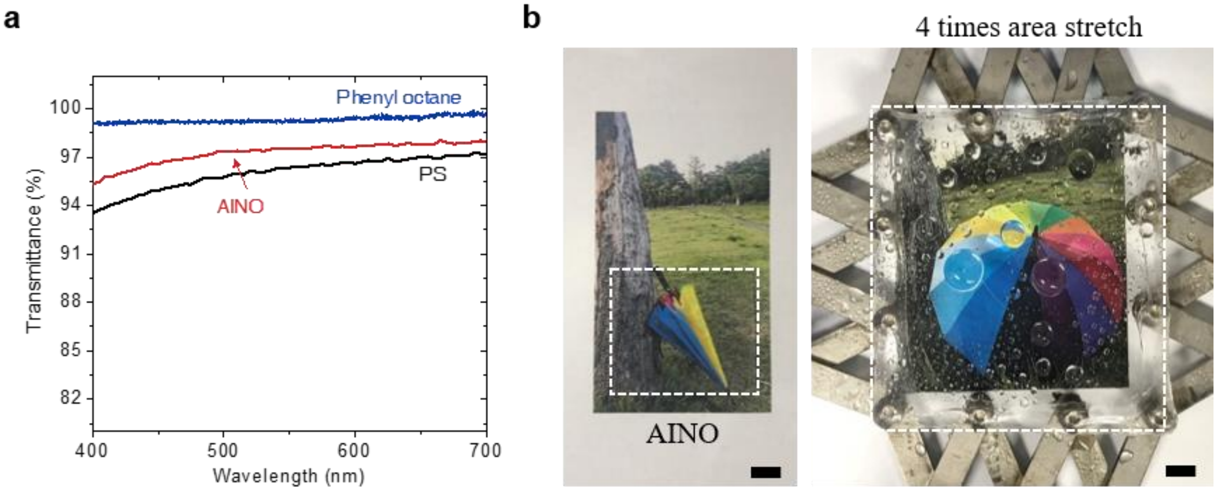

Supplementary Fig. 12. Transparency of the AINOs. a, Transmittance of the AINO and its components: PS and phenyl octane. The transmittance of the PS and AINO specimens is measured in film shape, whose thickness is 1 mm, with the ambient air as the reference. The transmittance of phenyl octane is measured in glass cuvette, whose thickness is 11 mm, with the deionized water as the reference. **b,** The AINO is transparent in the range of visible light, while still maintaining its transparency and hydrophobicity in biaxially stretched state by rigid frame. Scale bar = 1 cm.

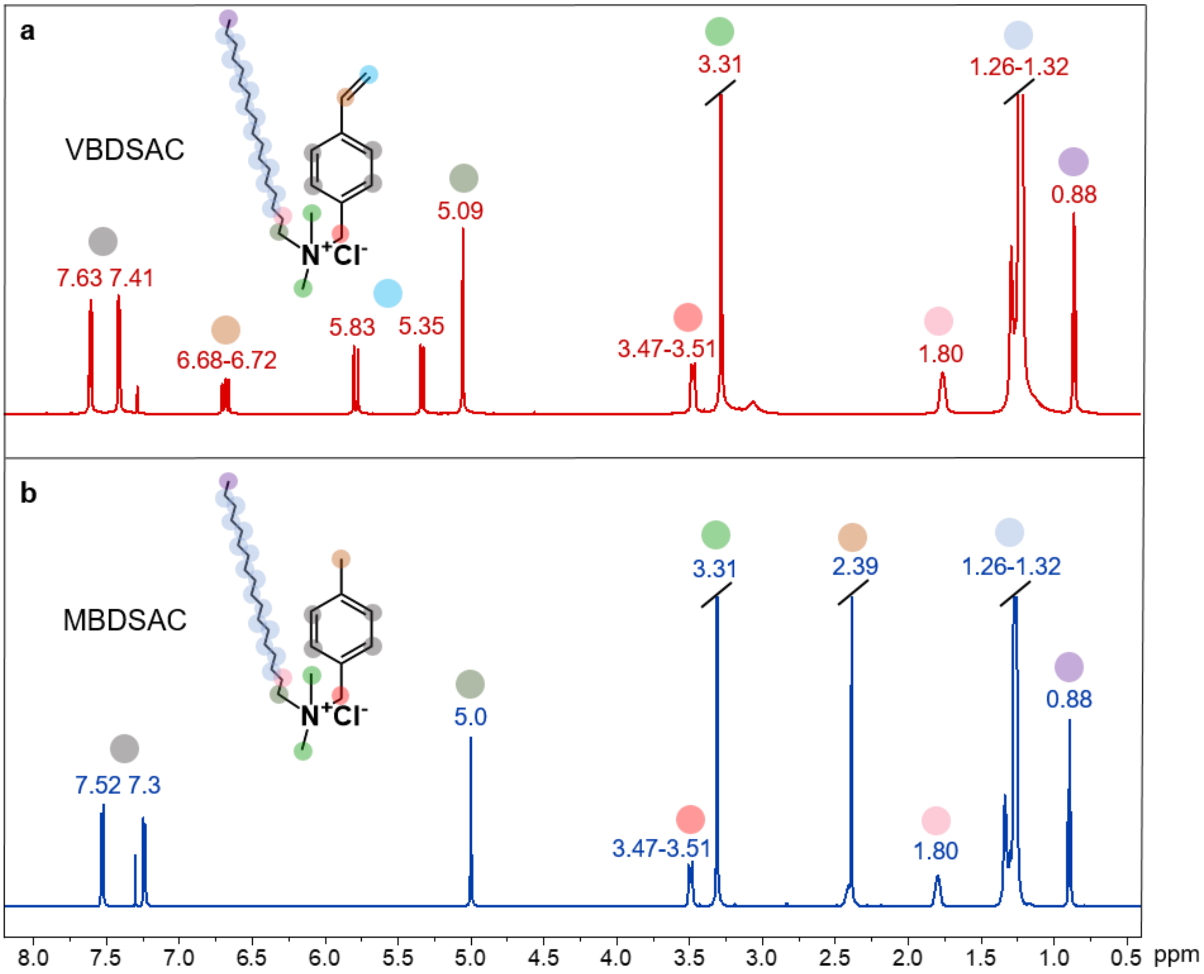

Supplementary Fig. 13. ^{1}H-NMR spectra of the synthesized capping ligands. a, ^{1}H-NMR spectra of 4-vinylbenzylstearylammonium chloride (VBDSAC). **b,** ^{1}H-NMR spectra of 4-methylbenzylstearylammonium chloride (MBDSAC).

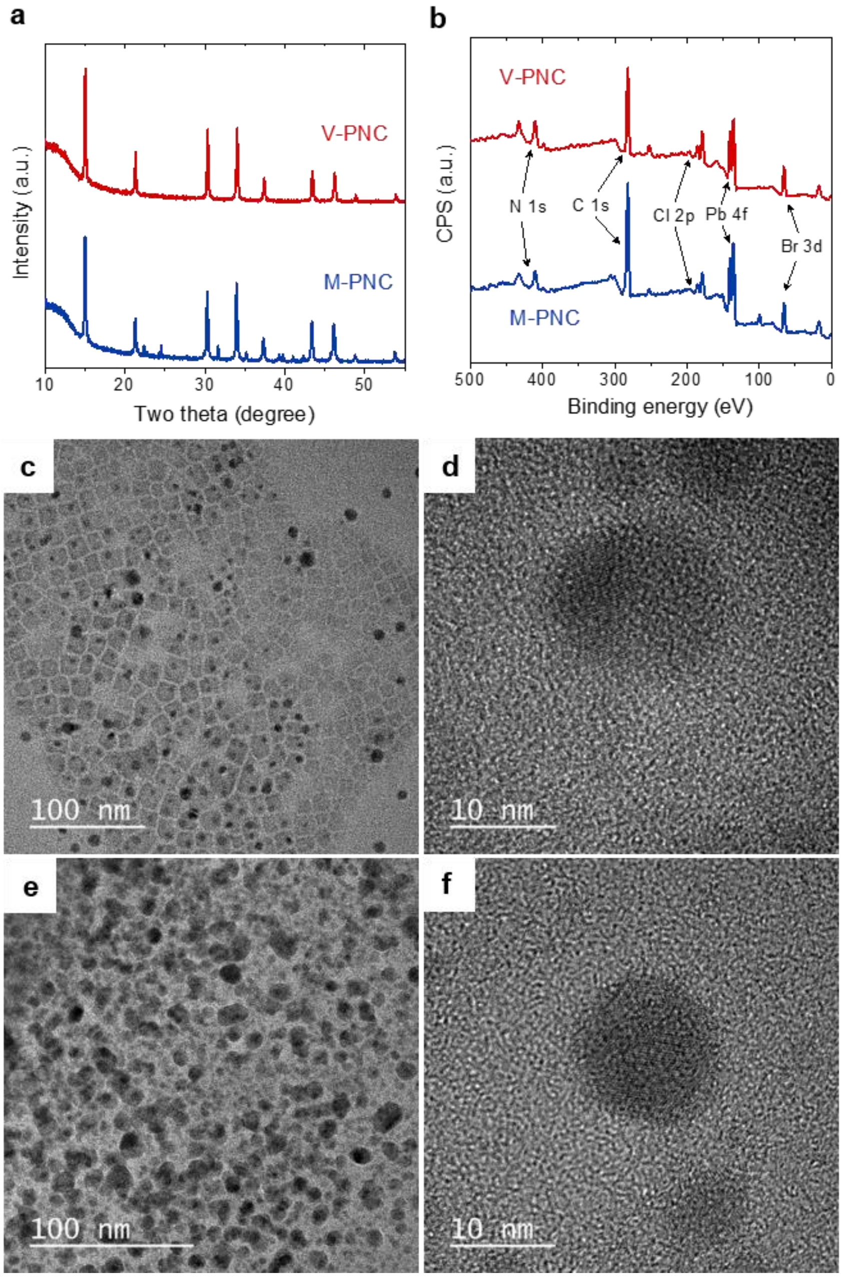

Supplementary Fig. 14. Characterizations of the synthesized PNCs. a, X-ray diffraction (XRD) results of the M-PNCs and V-PNCs. **b,** X-ray photoelectron spectroscopy (XPS) results of the M-PNCs and V-PNCs. **c-f,** Transmission electron microscopy (TEM) images of the V-PNCs (**c**) and higher magnification scale (**d**), and TEM images of the M-PNCs (**e**) and higher magnification scale (**f**).

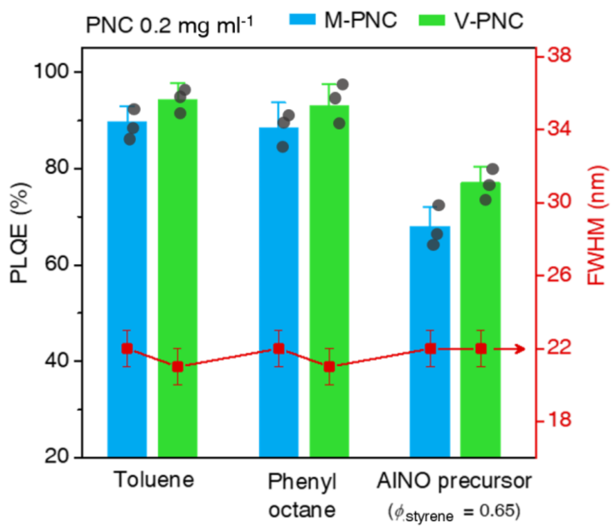

Supplementary Fig. 15. Effect of the solvent type on the luminescence properties of PNCs. PLQE and FWHM of the PNCs according to the type of nonpolar solvents. Both of the M-PNC and V-PNC have high PLQE (around 90 % for the M-PNC and 95 % for the V-PNC) when dispersed in toluene or phenyl octane. On the other hand, when the PNCs are dispersed in the AINO precursor, consisting of styrene monomer, divinylbenzene crosslinker, phenyl octane solvent, and AIBN free radical initiators, their PLQE drops compared to those in pure solvent. This luminescence degradation possibly originates from luminescence quenching by the radicals of the vinyl group in the monomer or radical initiator. Values represent the mean and standard deviation (n = 3).

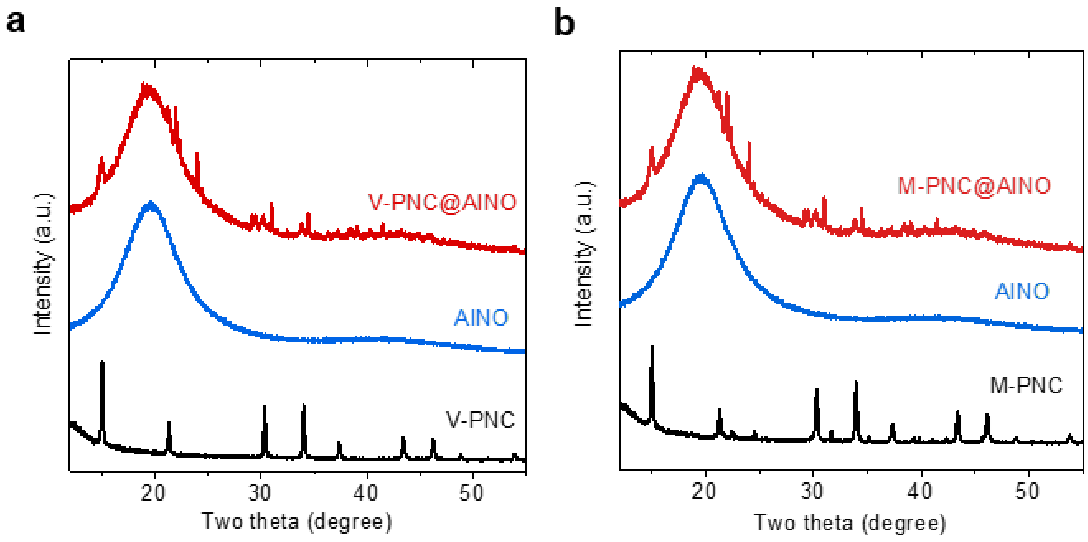

Supplementary Fig. 16. XRD results of the PNC, AINO and PNC@AINO. a, XRD spectra of the V-PNC, AINO, and V-PNC@AINO. **b,** XRD spectra of the M-PNC, AINO, and M-PNC@AINO. Solvent and polymer volume concentration of the AINO and V-PNC@AINO are phenyl octane and 0.65, respectively. The PNC concentration of the both nanocomposites is 12 mg ml^{-1}.

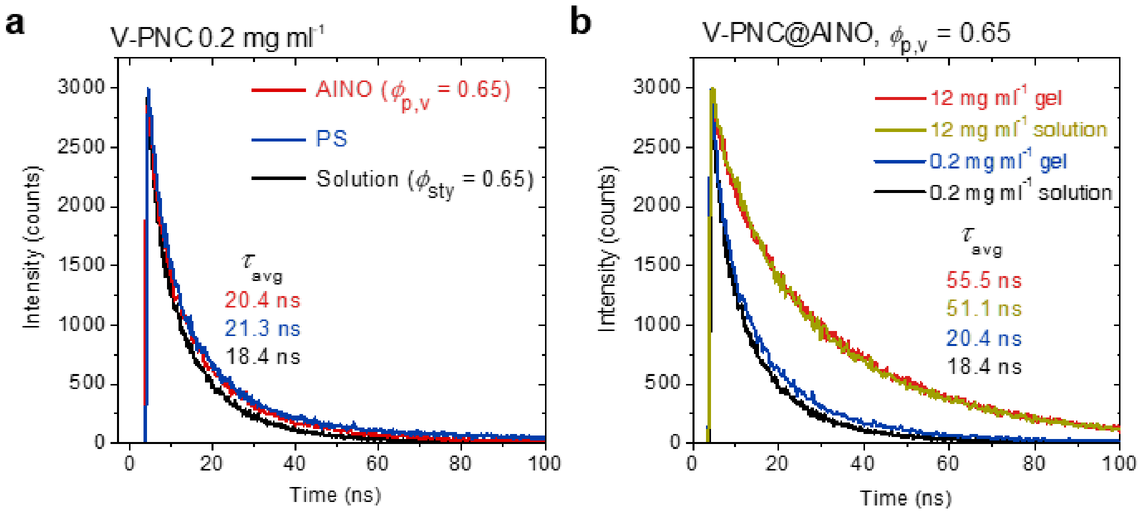

Supplementary Fig. 17. PL lifetime decays of the PNC nanocomposites. a, PL lifetime decays of the V-PNC nanocomposites according to the matrix state: liquid (precursor solution), rigid solid (PS), and gel (AINO). The PNC concentration is 0.2 mg ml^{-1}. **b,** PL lifetime decays of the V-PNC@AINO nanocomposites and precursor solutions with varying their PNC concentration. The average PL lifetime τ_{avg} is fitted by a di-exponential function and the equations followed: $A(t) = A_0 + A_1\exp\{-(t-t_0)\,\tau_1^{-1}\} + \mathrm{A}_2\exp\{-(t-t_0)\,\tau_2^{-1}\}$, and $\tau_{avg} = (A_1\tau_1^2 + A_2\tau_2^2)\,(A_1\tau_1 + A_2\tau_2)^{-1}$.

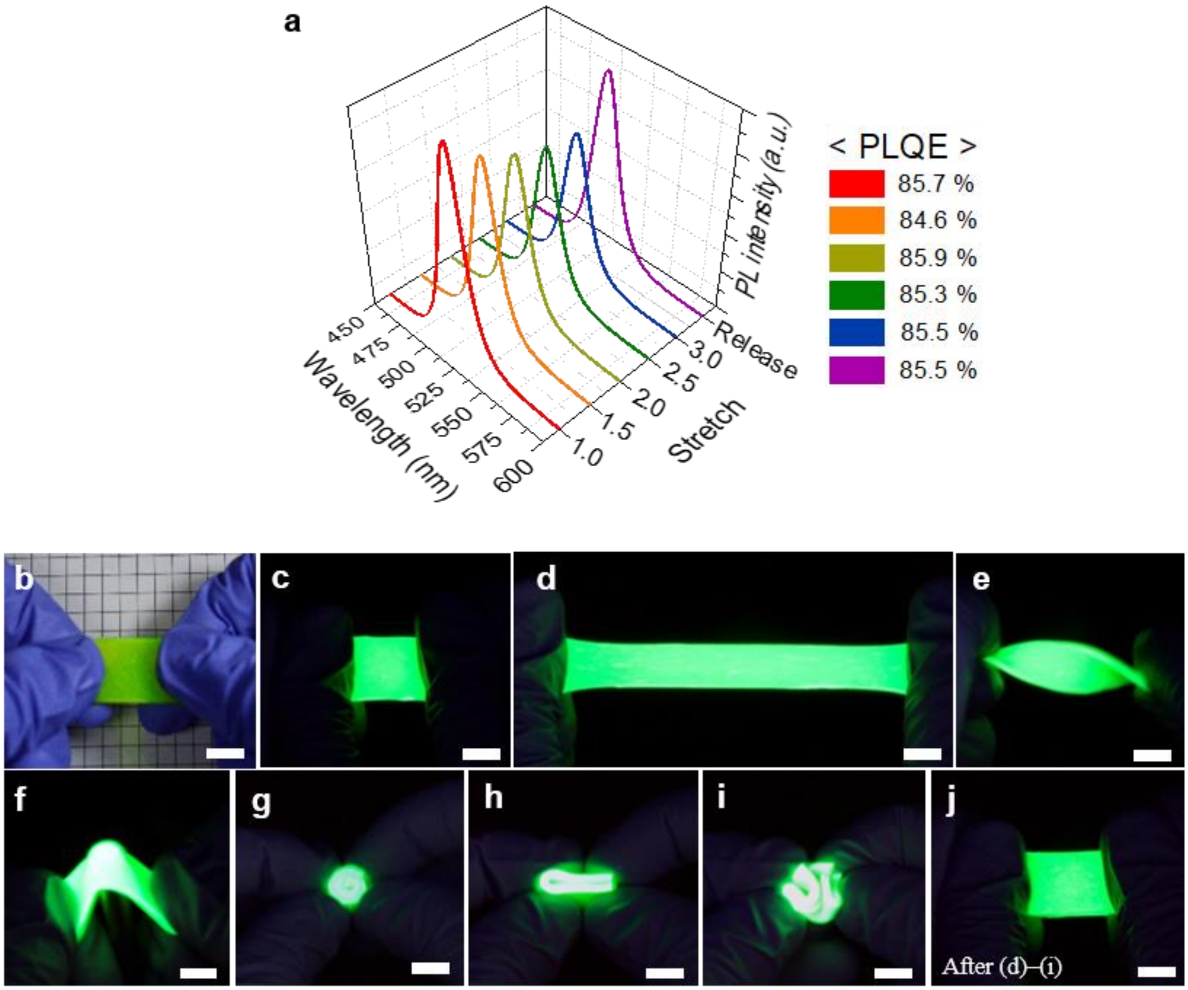

Supplementary Fig. 18. Luminescence of the V-PNC@AINO under deformation. a, List of PL spectra and corresponding PLQE values of the V-PNC@AINO, while gradually stretched to 3 times its original length and released. The PLQE values represent the mean (n = 3–5). **b-j,** Photographs of the bright green luminescence of the nanocomposite undergoing versatile deformation, from its pristine state in daylight (**b**) and under UV irradiation (**c**) to a series of deformations, including stretching (**d**), twisting (**e**), poking (**f**), rolling (**g**), folding (**h**), crumpling (**i**). The nanocomposite still retains its bright luminescence after all kinds of deformation (**j**). Scale bar = 1 cm.

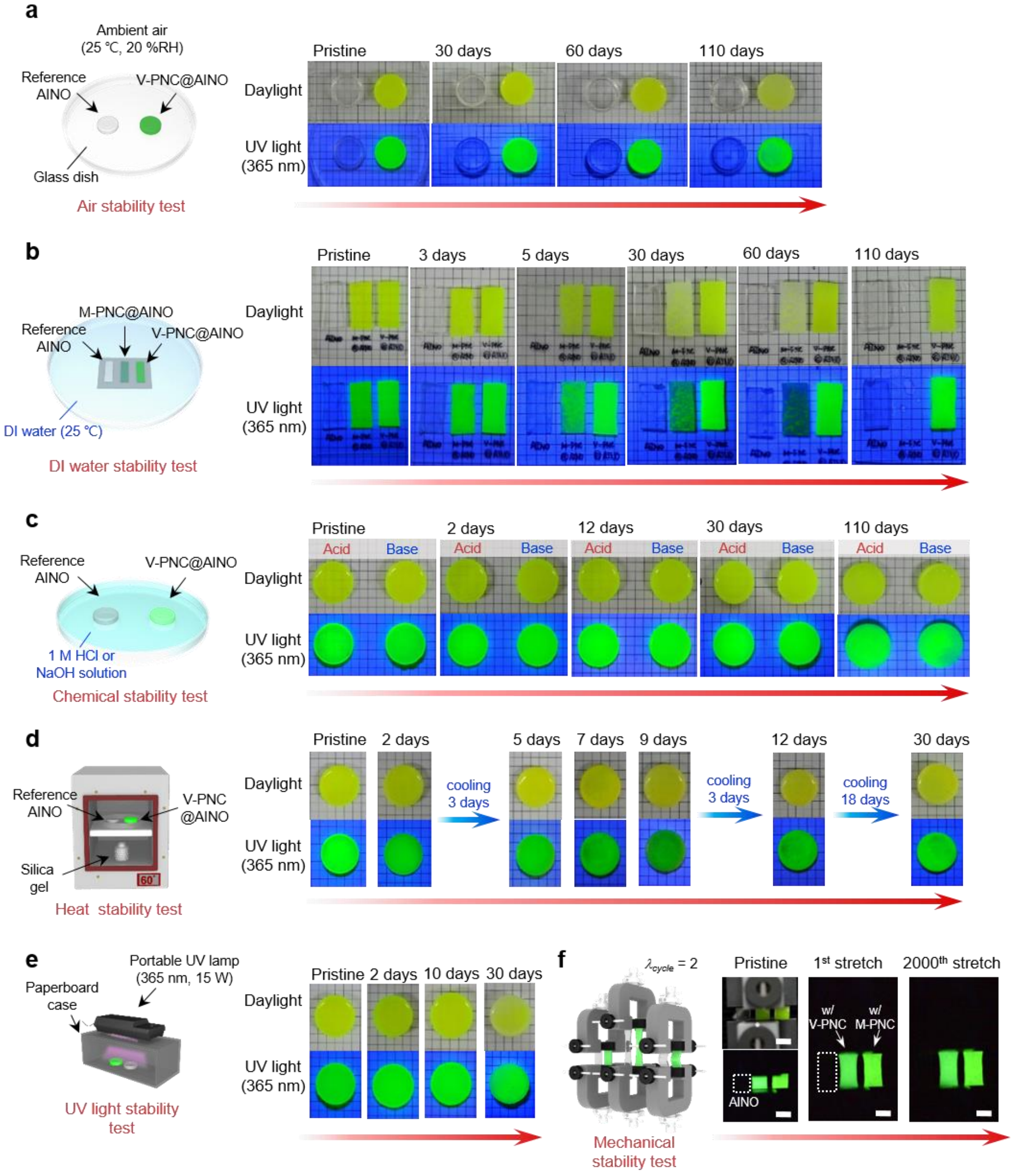

Supplementary Fig. 19. Environmental stability tests of the PNC@AINOs in length of time. **a-f,** Schematic design of the experimental setup and photographs of the nanocomposites during air stability test (**a**), water stability test (**b**), acid and base stability test (**c**), heat stability test (**d**), UV light stability test (**e**), and mechanical stability test (**f**). The AINOs without PNCs are located together with the nanocomposites in all of the stability tests as the references. The length of a single square grid in the photographs of **a**–**e** is 0.5 cm. Scale bar in **f** = 1 cm.

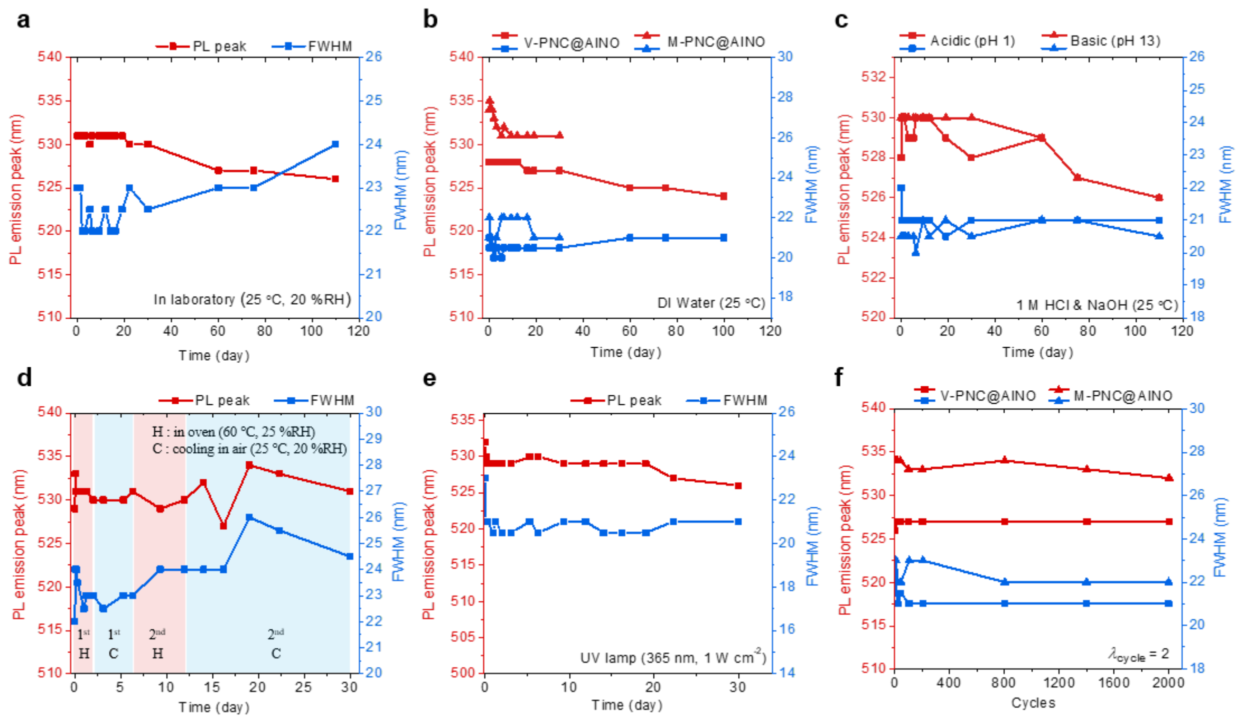

Supplementary Fig. 20. PL emission peak and FWHM during environmental stability tests. **a,** PL emission peak and FWHM of the V-PNC@AINO during air stability test. **b,** PL emission peak and FWHM of the PNC@AINOs during DI water stability test. **c,** PL emission peak and FWHM of the V-PNC@AINO during acid and base stability test. **d,** PL emission peak and FWHM of the V-PNC@AINO during heat stability test. **e,** PL emission peak and FWHM of the V-PNC@AINO under UV irradiation. **f,** PL emission peak and FWHM of the PNC@AINOs during stretch-and-release cycle test. The concentration of PNC in all of the nanocomposites for stability tests is 12 mg ml^{-1}.

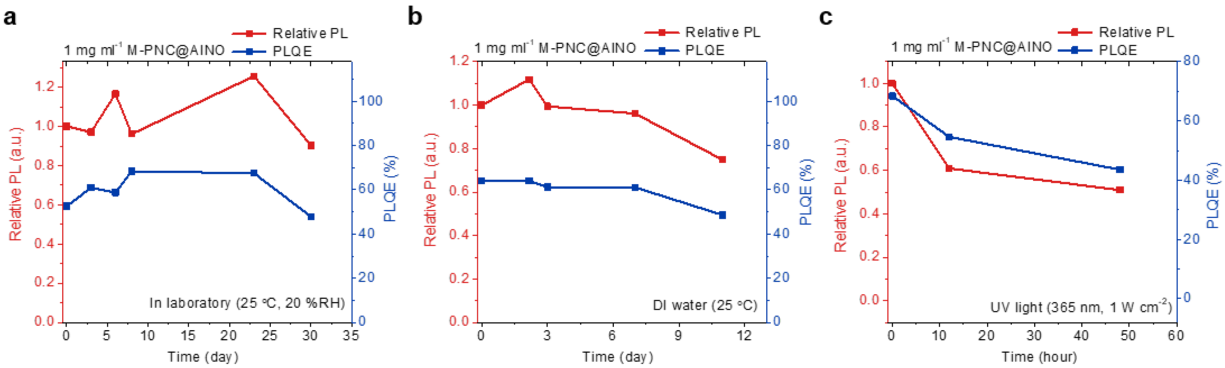

Supplementary Fig. 21. Environmental stabilities of the M-PNC@AINOs. a, Relative PL intensity and PLQE values of the M-PNC@AINOs in ambient air. **b,** Relative PL intensity and PLQE values of the M-PNC@AINOs in DI water. **c,** Relative PL intensity and PLQE values of the M-PNC@AINOs under UV irradiation.

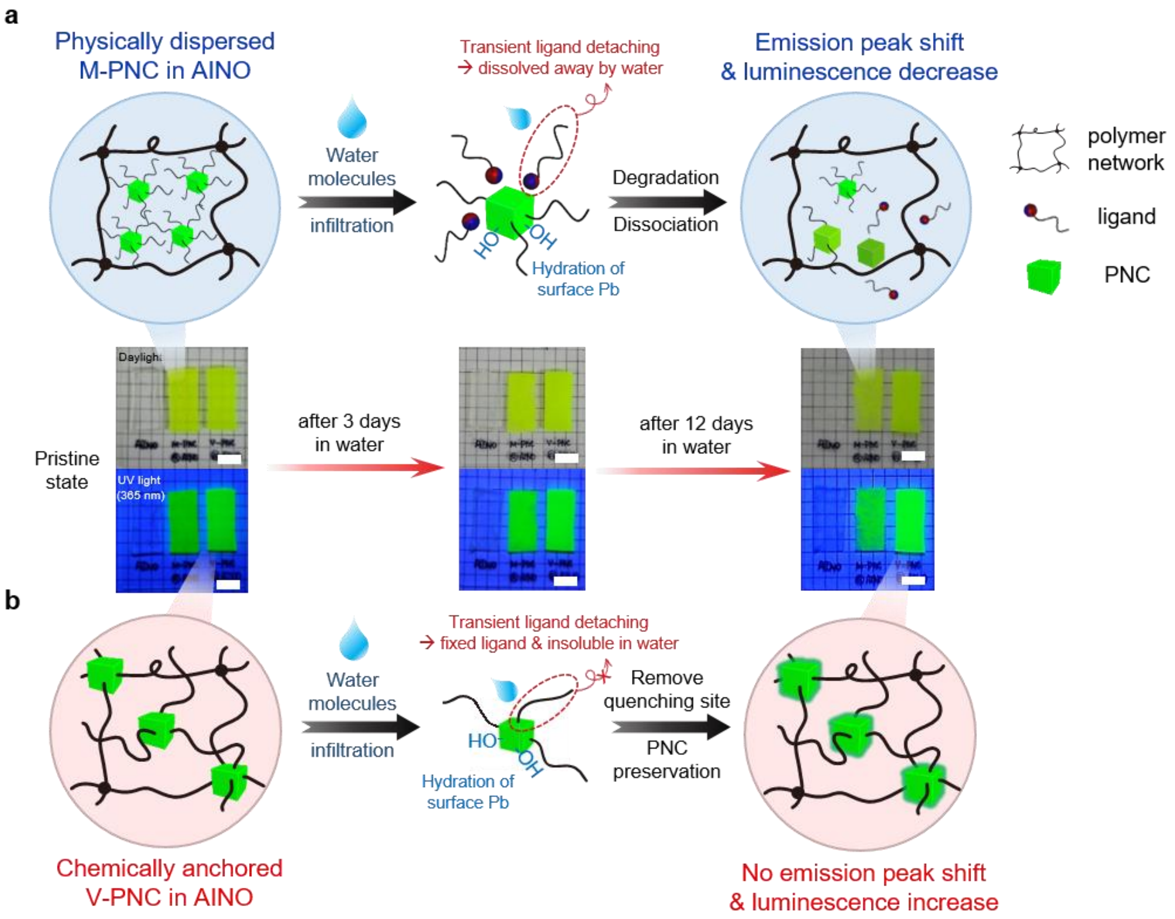

Supplementary Fig. 22. Explanation of the different luminescence behaviors in water. a, When the M-PNC@AINO, whose PNCs are physically dispersed in the AINO, is soaked into water, the infiltrated water molecules hydrate the surface Pb of the PNC, and detach the ionic-bonded ligands from the PNCs, and then dissolve the ligands away. Therefore, the PL intensity of the M-PNC@AINO increases in initial state due to removal of the surface metallic Pb, which acts as luminescence quenching sites, but the intensity gradually decreases with emission peak shift, which originates from the dissociation or decomposition of the PNCs by the water molecules. **b,** In contrast, when the V-PNC@AINO, whose PNCs are chemically anchored in the AINO, is soaked into water, the infiltrated water molecules hydrate the surface Pb likewise, and detach the ionic-bonded ligands from the PNCs transiently, but the water molecules cannot dissolve away the ligands, as they are anchored to the nonpolar polymer chains. Therefore, the PL intensity of the V-PNC@AINO is increased and maintained in the water for more than 60 days, with no emission peak shift. Scale bar = 1 cm.

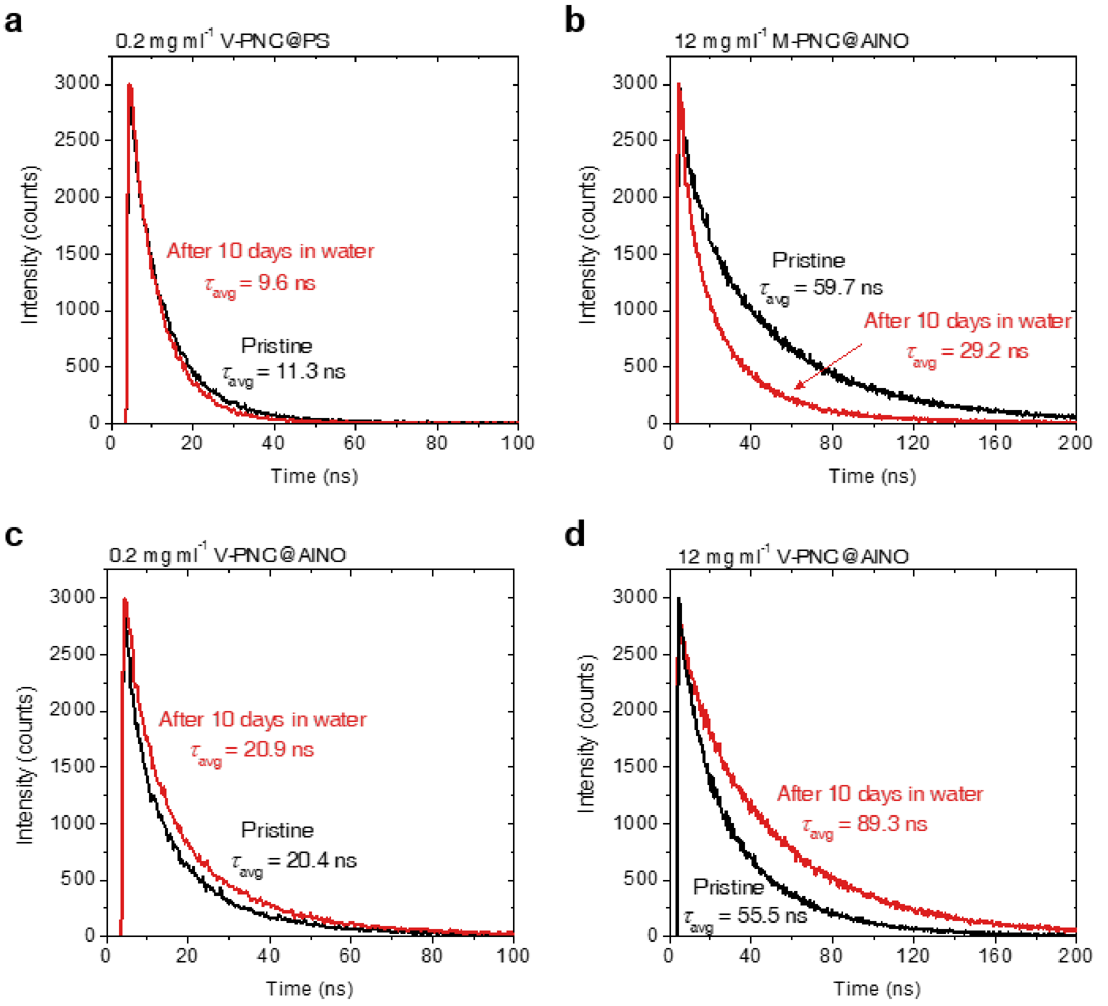

Supplementary Fig. 23. PL lifetime decay behaviors of the PNC nanocomposites in water. a, PL lifetime decays of the V-PNC@PS nanocomposite in initial state and after 10 days in water. The water molecules hardly infiltrate into the PS, resulting in the little change in its average PL lifetime τ_{avg}. **b,** PL lifetime decays of the M-PNC@AINO nanocomposite with saturated concentration of the PNC in the initial state and after 10 days in water. The infiltrated water molecules markedly decompose the PNCs in the nanocomposite, resulting in the decrease of its τ_{avg}. **c,d,** PL lifetime decays of the V-PNC@AINO nanocomposites with low PNC concentration (**c**) and saturated PNC concentration (**d**) in the initial state and after 10 days in water. The infiltrated water molecules hydrate the surface metallic Pb of the PNC, resulting in the increase of its τ_{avg} by lowering the surface defect of PNCs. The τ_{avg} is fitted by a di-exponential function and the equations followed; $A(t) = A_0 + A_1\exp\{-(t\text{-}t_0)\ \tau_1^{-1}\} + \mathrm{A}_2\exp\{-(t\text{-}t_0)\ \tau_2^{-1}\}$, and $\tau_{avg} = (A_1\tau_1^2 + A_2\tau_2^2)\ (A_1\tau_1 + A_2\tau_2)^{-1}$

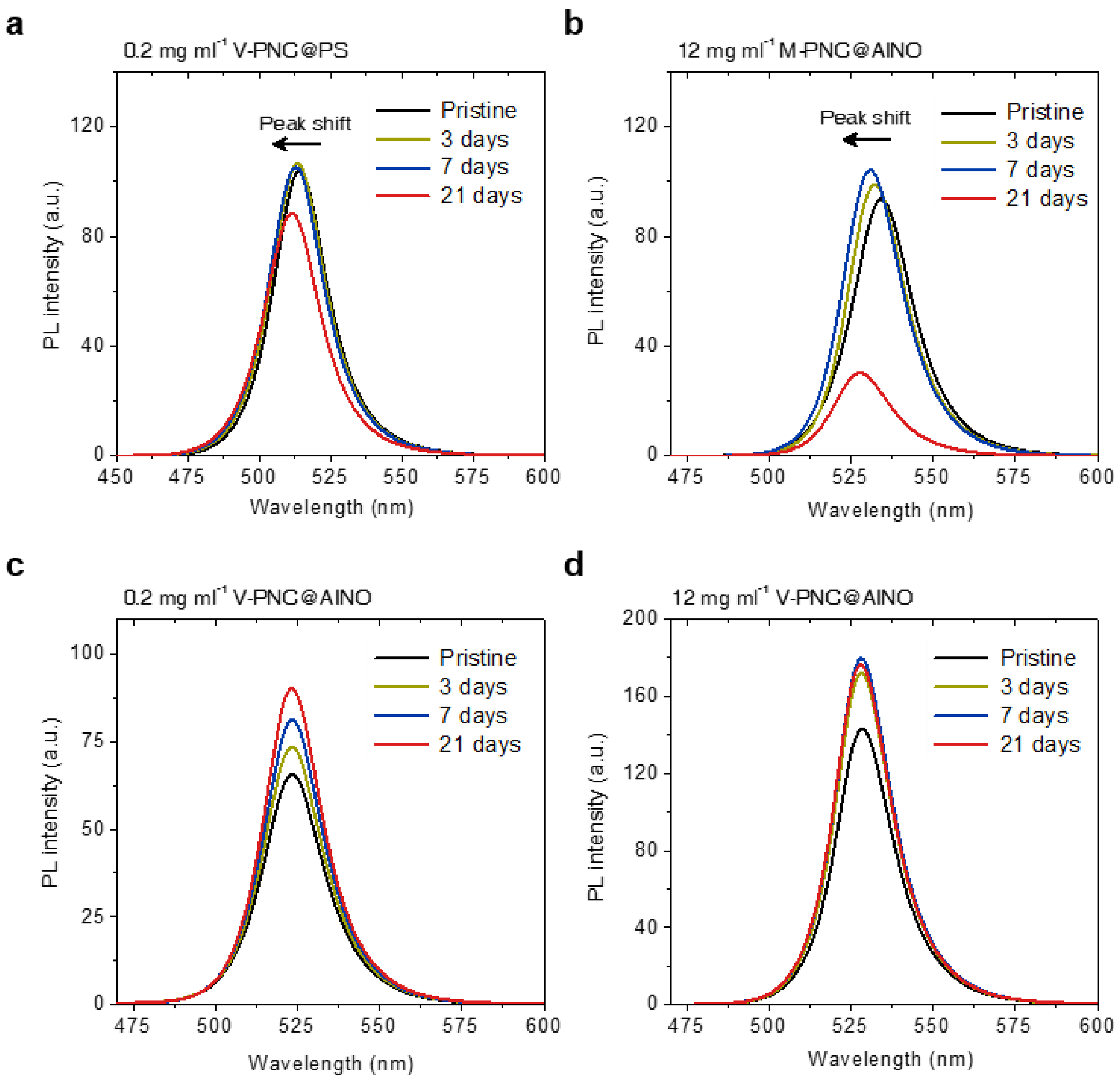

Supplementary Fig. 24. PL behaviors of the PNC nanocomposites in water. a, PL spectra of the V-PNC@PS soaked in water for 21 days. **b,** PL spectra of the M-PNC@AlNO with saturated concentration soaked in water for 21 days. The PL intensity of the nanocomposites increases in the first 7 days, but decreased markedly after 21 days with blue shift of the emission peak. **c, d,** PL spectra of the V-PNC@AlNO with low PNC concentration (**c**) and saturated PNC concentration (**d**) soaked in water for 21 days. The PL intensity of the V-PNC@AlNOs increases with length of time, with no emission peak shift for 21 days.

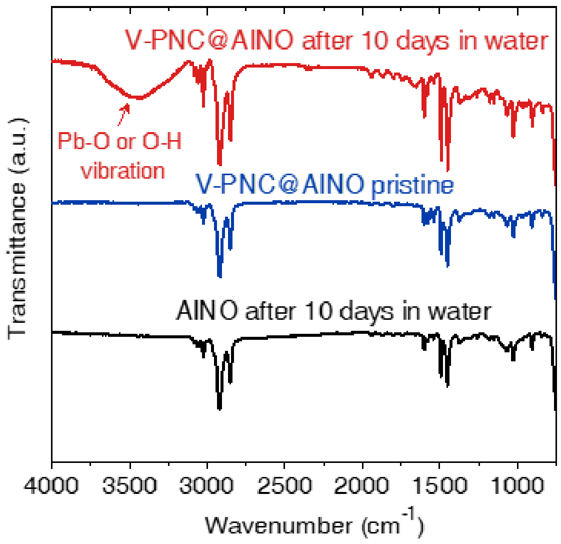

Supplementary Fig. 25. FT-IR spectra of the AINO and nanocomposite in water. The V-PNC@AINO after 10 days in water has clear broad peak observed in the range 3500 cm^{-1}, which peak is assigned to Pb-O and O-H stretching vibrations.

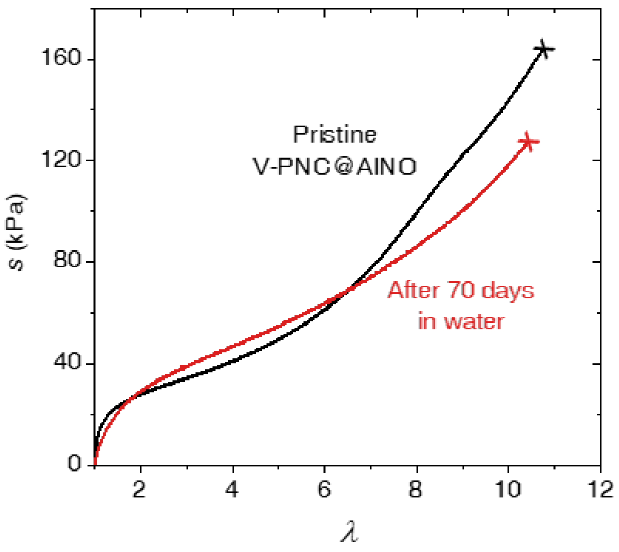

Supplementary Fig. 26. Water effect on the mechanical behavior of V-PNC@AINO. Mechanical tensile stress–stretch curves of the V-PNC@AINOs from the initial state and after 70 days in water. Solvent, polymer volume concentration, and PNC concentration of the nanocomposite are phenyl octane, 0.65, and 12 mg ml^{-1}, respectively.

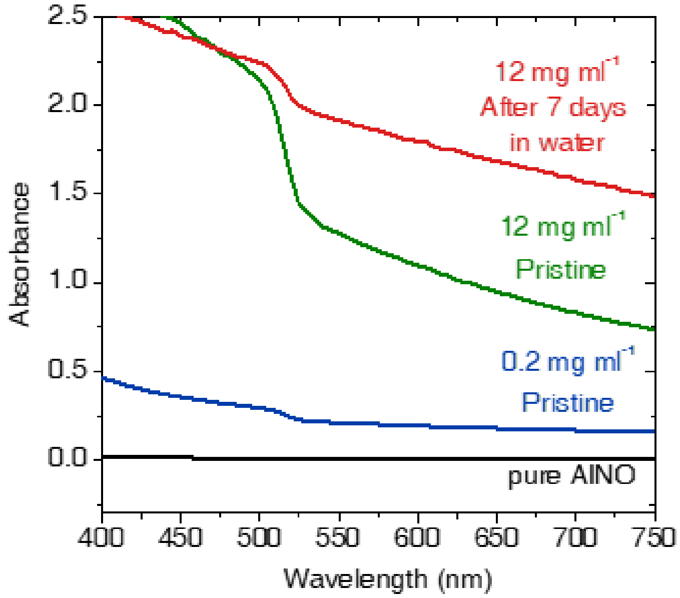

Supplementary Fig. 27. Effect of PNC concentration and water soaking on absorbance. Absorption spectra of the pure AINO, and the V-PNC@AINO with low PNC concentration (0.2 mg ml^{-1}) and saturated PNC concentration (12 mg ml^{-1}), and the V-PNC@AINO with saturated concentration after immersion in water for 7 days. Since the high PNC concentration and water soaking increase the absorbance of the luminescent nanocomposite, which makes color conversion efficiently, we use the V-PNC@AINO with saturated PNC concentration of 12 mg ml^{-1}, soaking in water for 7 days, as a color conversion layer of fully deformable pure green light-emitting devices.

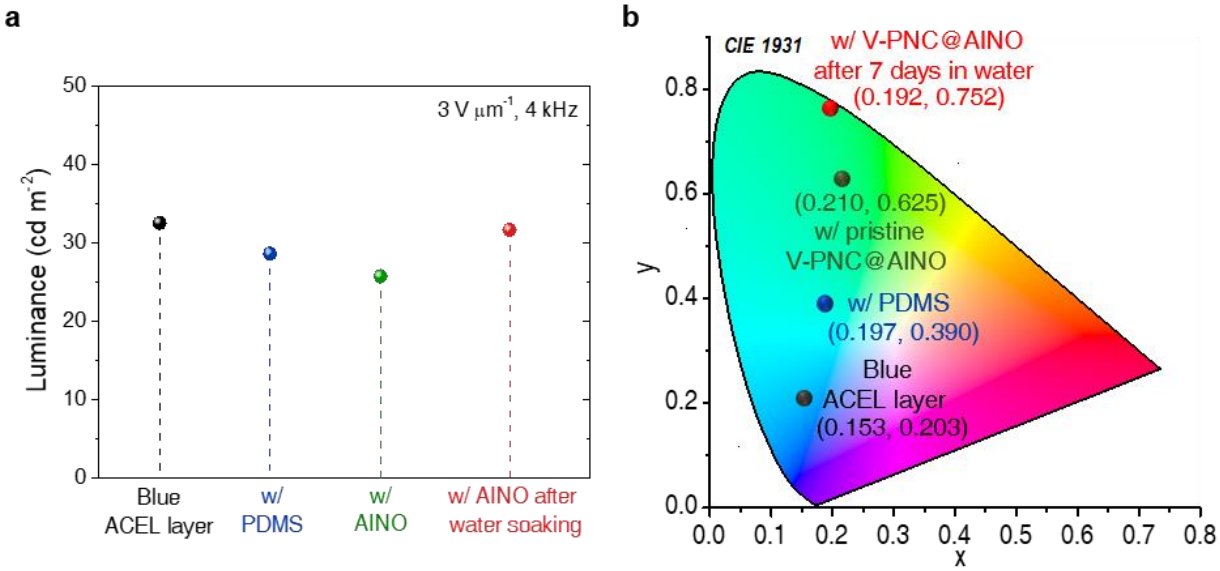

Supplementary Fig. 28. Water effect on the V-PNC@AINO as a color conversion layer. a, Luminance of the devices with different color conversion layer, including pristine blue ACEL layer, V-PNC with PDMS (V-PNC@PDMS), pristine V-PNC@AINO, and V-PNC@AINO soaked in water for 7 days. The applied electric field and frequency is 3 V μm^{-1} and 4 kHz, respectively. **b,** Color coordinates of the devices with different color conversion layer, including pristine blue ACEL layer, V-PNC with PDMS (V-PNC@PDMS), pristine V-PNC@AINO, and V-PNC@AINO soaked in water for 7 days.

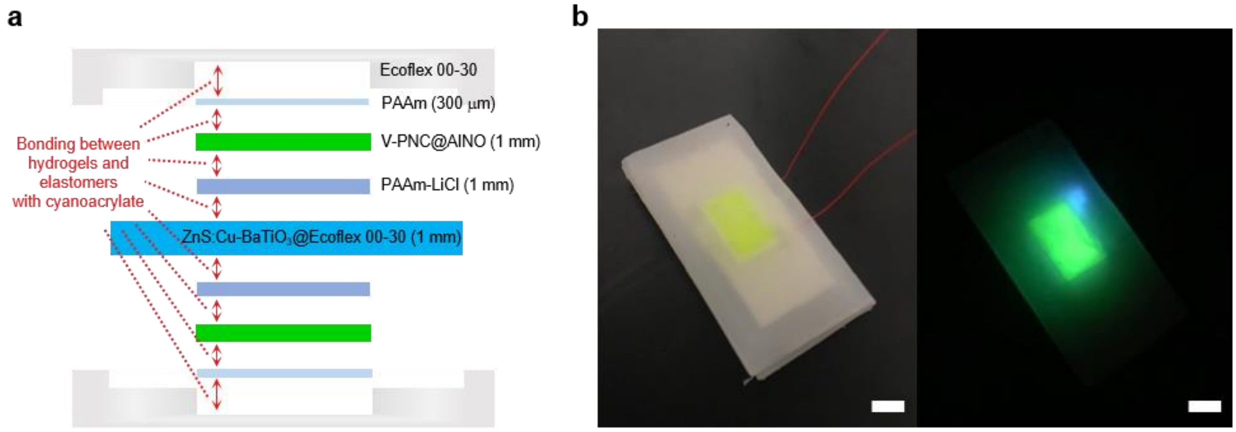

Supplementary Fig. 29. Structure of fully deformable pure green light-emitting devices. a, Schematic design of the device structure. The interfaces between each hydrogel and elastomer layer are bonded by the reported method using diluted cyanoacrylate adhesives[30]. PAAm, polyacrylamide hydrogel; PAAm-LiCl, PAAm hydrogel ionic conductor with lithium chloride salt. The total thickness of the devices is about 7 mm. **b,** Photographs of the fabricated device under daylight (left) and under operation in dark room (right). Scale bar = 1 cm.

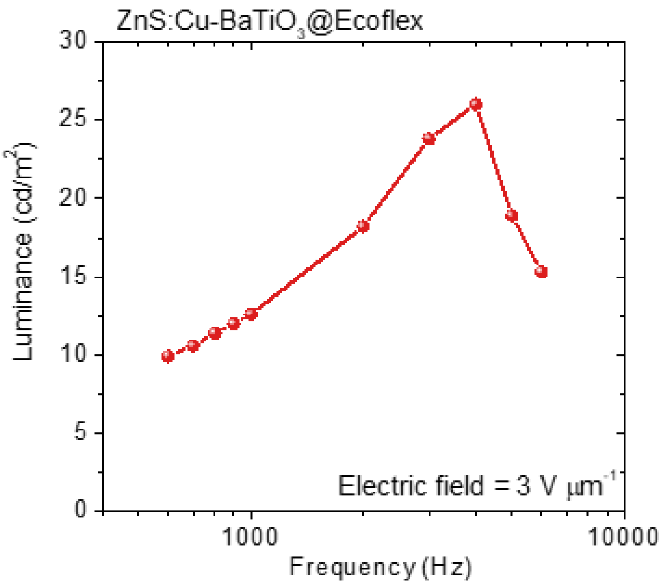

Supplementary Fig. 30. Luminance of blue ACEL layer as a function of the frequency. Luminance of the blue ACEL layer, composed of two hydrogel ionic conductors and ZnS:Cu-$BaTiO_3$@Ecoflex electroluminescent nanocomposite, as a function of the frequency of applied AC voltages. When the frequency is low, the power of electric field is insufficient to excite the ACEL particles, whereas when the frequency is too high, the electrons and holes cannot respond in the ACEL particles[3]. The ACEL particles used in this work show highest luminance at 4 kHz, so we fix the frequency of AC voltages to 4 kHz for the device operation.

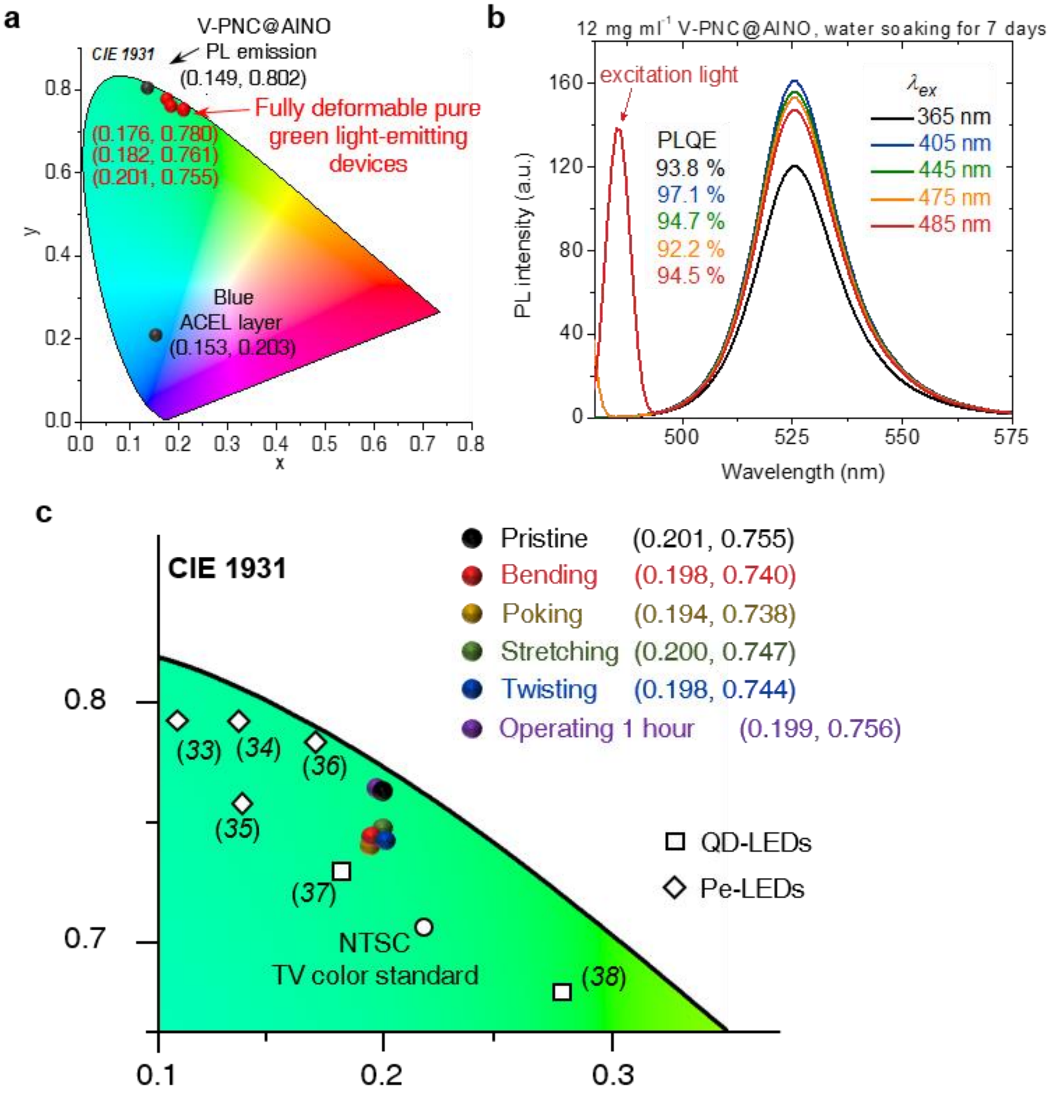

Supplementary Fig. 31. Luminescence properties of the devices. a, Color coordinates of PL emission of the color conversion layer (V-PNC@AINO), electroluminescence of the blue ACEL layer (ZnS:Cu-$BaTiO_3$@Ecoflex), and electroluminescence of the device on the CIE 1931 color space. **b,** PL spectra and PLQE values of the V-PNC@AINO soaked in water for 7 days as a color conversion layer, which is excited by the wavelength of blue light series from 365 to 485 nm. **c,** Color coordinates of the device in various deformed state and after 1 hour operation, plotted together with color coordinates of the NTSC TV color standard, and reported studies of Pe-LEDs[33,34,35,36] and QD-LEDs[37,38] on the CIE 1931 color space.

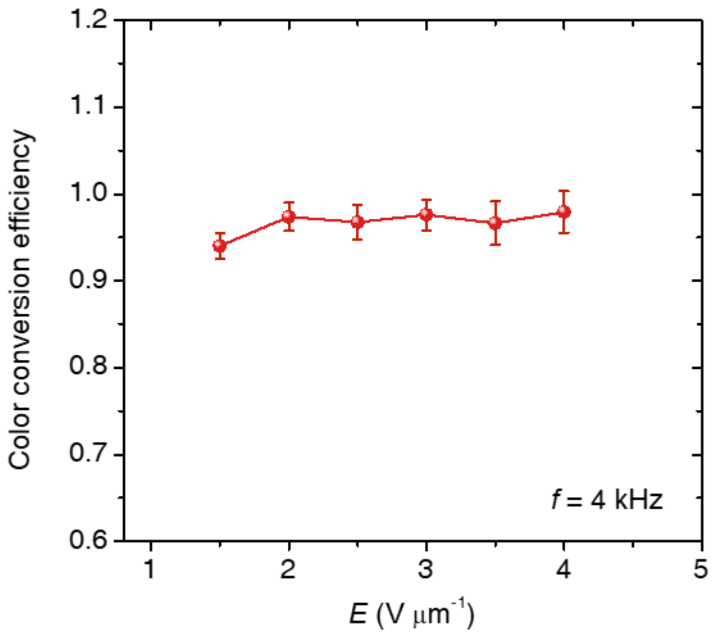

Supplementary Fig. 32. Color conversion efficiency of the devices. The color conversion efficiency is calculated by dividing the luminance of the device with the V-PNC@AINO layer into the luminance of the device without the V-PNC@AINO layer.

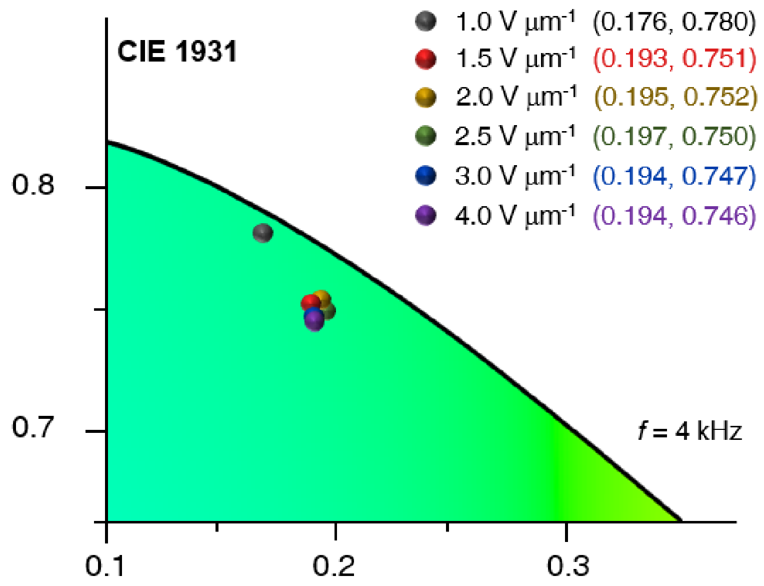

Supplementary Fig. 33. Effect of electric field on the color coordinate of the device. Color coordinates of the device on the CIE 1931 color space according to the applied AC electric field. When the electric field is too low (1 V μm^{-1}), the color purity is high but dim (the luminance is below 1 cd m^{-2}). For the electric fields showing enough bright luminescence (above 1.5 V μm^{-1}), the color purity is not much relevant with the intensity of applied electric field, where the color coordinates of the device are near (0.2, 0.75). The frequency of applied voltage is 4 kHz.

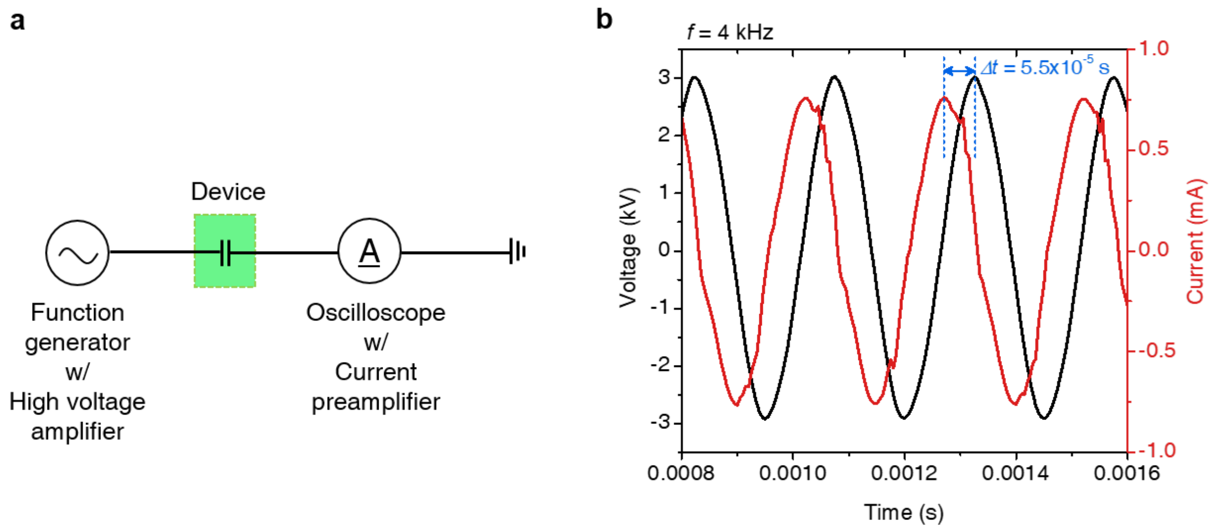

Supplementary Fig. 34. Power consumption of the device. a, Electric circuit for the measurement of power consumption of the device. The voltage is applied to the device by the function generator with the high voltage amplifier, while the current and the phase shift is measured by the oscilloscope with the current preamplifier. **b,** Applied voltage and the measured current as a function of time. The applied frequency and the voltage is 4 kHz and 6 kV_{pp}, respectively. The time difference at peak position of the voltage and the current is $5.5x10^{-5}$ s, therefore the phase shift is calculated to 79.2º. The power consumption is 0.21 W, obtained by the following equation, $P = V_{RMS}I_{RMS}cos\phi$, where V_{RMS} and I_{RMS} are the voltage and the current of root-mean-square value, and $cos\phi$ is the phase shift.

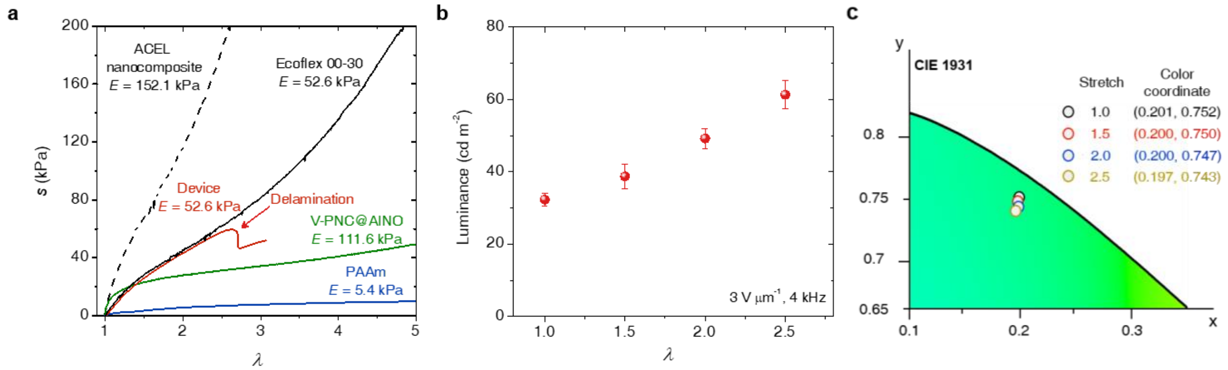

Supplementary Fig. 35. Display performance of the device with mechanical properties. a, Tensile stress-stretch curves of the integrated device and its constituents, including PAAm hydrogel conductor, V-PNC@AINO, ACEL nanocomposite (ZnS:Cu-$BaTiO_3$@Ecoflex), and encapsulating matrix Ecoflex 00-30. The interface between ACEL nanocomposite and encapsulating matrix is delaminated when the stretch reaches about 2.7. **b,** Luminance of the device as a function of applied stretch. Values represent the mean and standard deviation (n = 3). **c,** Color coordinates of the emission light of the device under stretch. The applied voltage and frequency is 3 kV and *4* kHz, respectively.

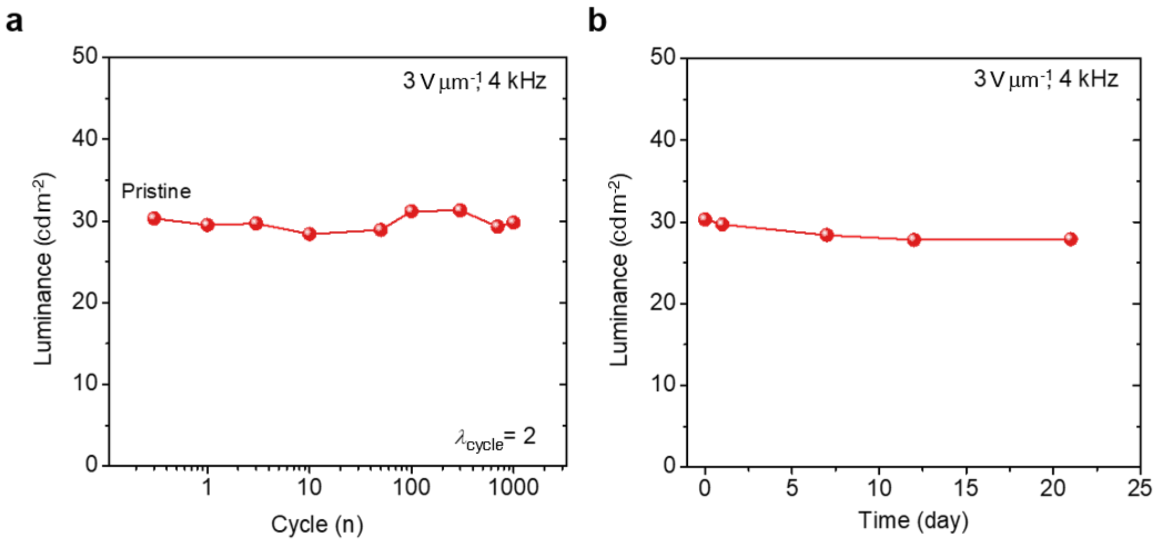

Supplementary Fig. 36. Durability of the device. a, Luminance of the device after cyclic stretch-release test. The cyclic stretch is 2. **b,** Luminance of the device with time interval in ambient air. The applied electric field and frequency for both of the durability tests are fixed to 3 V μm^{-1} and 4 kHz, respectively.

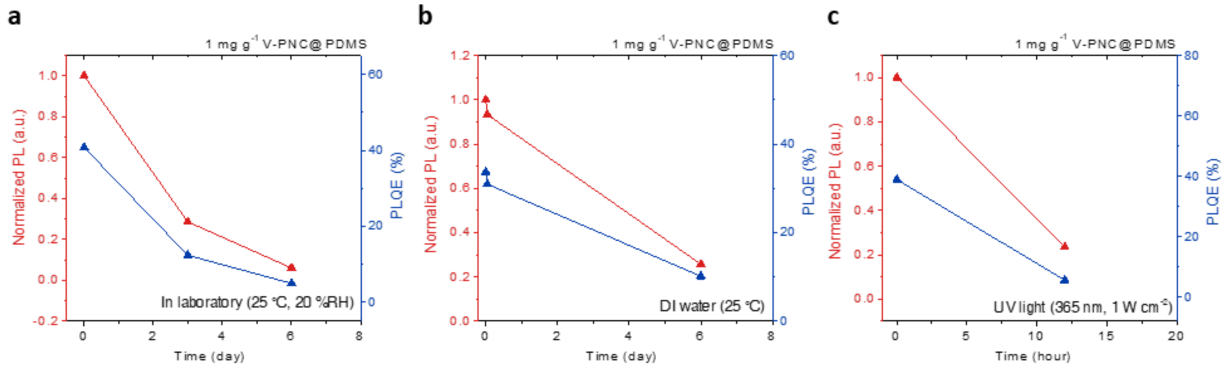

Supplementary Fig. 37. Environmental stability of the V-PNC@PDMS nanocomposites. a, Relative PL intensity and PLQE of the V-PNC@PDMS in ambient air. **b,** Relative PL intensity and PLQE of the V-PNC@PDMS in DI water. **c,** Relative PL intensity and PLQE of the V-PNC@PDMS under UV irradiation.

Components			ΔH_v (kJ mol^{-1})	V_m (ml mol^{-1})	δ (MPa$^{1/2}$)	Melting point (℃)	Boiling point (℃)
Type	Name	Chemical structure					
Polymers	PS	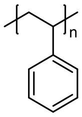			18.5		
	PMMA	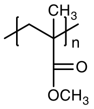			19.0		
Solvents	Hexane	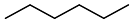	31.0	131.6	14.72	-95	68
	Decane	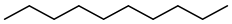	51.3	194.9	15.8	-30	174
	Methyl cyclohexane	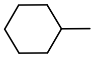	35.3	127.5	16.0	-126	101
	Butyl cyclohexane	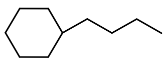	49.0	171.5	16.5	-78	180
	γ-Terpinene	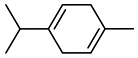	51.4	159.7	17.5	-10	183
	Terpinolene	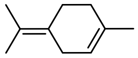	50.8	159.6	17.3	-	184
	Toluene	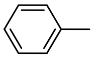	37.0	112.9	17.5	-95	110
	Phenyl butane	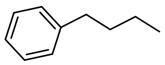	50.8	156.1	17.7	-88	183
	Phenyl hexane	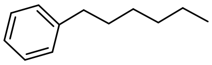	60.4	188.5	17.5	-61	226
	Phenyl octane	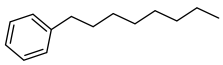	68.1	221.8	17.2	-36	265
	Phenyl dodecane	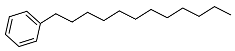	86.6	287.88	17.1	-3	320

Supplementary Table 1. Thermodynamic parameters of the materials used in this study. The vaporization enthalpy ΔH_v, molar volume V_m, melting point, and boiling point are obtained from the National Institute of Standards and Technology (NIST) and the preceding studies[39,40,41,42]. The solubility parameter of the solvents is calculated from Supplementary equation (1), and the solubility parameter of the polymers are quoted from the reference[43].

Reference DI water	M-PNC@AINO after 14 days	V-PNC@AINO after 14 days	V-PNC@AINO after 70 days
ND	0.162 ppm	ND	0.15 ppm

Supplementary Table 2. Release content of Pb from the PNC@AINOs. ICP-AES results for defining content of Pb in water released from the PNC@AINO specimens during water stability test. The resolution of the spectrometer is 0.4 ppb. The abbreviation ND means that Pb is not det ected by the spectrometer.

Supplementary Notes

Thermodynamic theories

1. Solubility parameter. When two different molecules are mixed, the preference of mixing can be explained by introducing the solubility parameter δ derived from the vaporization enthalpy ΔH_v and molar volume V_m of molecules[42]:

$$\delta = \sqrt{\frac{\Delta H_v - RT}{V_m}} \tag{1}$$

where T is temperature, and R is gas coefficient. Since it represents the strength of attraction between molecules in the unit volume, a smaller difference of δ between the molecules indicates their preferred mixing. Supplementary Table 1 shows the solubility parameter of the solvents δ_s used in this study, calculated from Supplementary equation 1. Since polymers generally decompose before evaporation, the solubility parameter of the polymers δ_p is not able to be calculated, but can be approximated empirically. Thus, the δ_p values of PS and PMMA used in this study are quoted from previous study[43].

Supplementary equation 1 is only valid for positive values of ΔH_v, meaning that specific effects, such as hydrogen bonding or charge complex interaction between the molecules, that can lead to negative values of the ΔH_v should be negligible to use the equation. Since we confine the type of solvents used in this study to highly nonpolar hydrocarbons, we use the above equation to calculate the solubility parameter for several solvents with no additional modifications. According to the reported theories[42], the solubility parameter can be defined by combining all of the solubility parameters affecting the intermolecular interaction between molecules, such as the Van der Waals dispersion force $\delta_{dispersion}$, dipole-dipole interaction δ_{dipole}, and hydrogen bonding $\delta_{hydrogen}$;

$$\delta_{tot}^2 = 4\delta_{dispersion}^2 + \delta_{dipole}^2 + \delta_{hydrogen}^2 \tag{2}$$

where the factor 4 in front of the dispersion solubility parameter is an empirically modified value. It would be desirable to use Supplementary equation (2) to characterize more polar systems.

2. Polymer-solvent interaction parameter. When it comes to the polymer–solvent system, the mixing of polymer and solvent is considered the contribution of both entropy change ΔS_{M} and enthalpy change ΔH_{M} [22]. The entropic contribution ΔS_{M} is statistically derived as:

$$\Delta S_{\mathrm{M}} = -k(N_{\mathrm{p}} \ln \phi_{\mathrm{p,v}} + N_{\mathrm{s}} \ln \phi_{\mathrm{p,s}}) \tag{3}$$

where k is the Boltzmann constant, N_{p} and N_{s} are the number of polymer segments and solvent, respectively, while $\phi_{\mathrm{p,v}}$ and $\phi_{\mathrm{p,s}}$ are the volume fractions of polymer and solvent, respectively. Note that N_{p} is ideally considered the number of monomer molecules in the polymer segments. In addition to the entropic effect of mixing, the enthalpy change ΔH_{M} is also considered the energy change during mixing, which undergoes the breakage of the interaction between the polymer and the solvent with each other, and the formation of interaction between the polymer and the solvent, written as:

$$\Delta H_{\mathrm{M}} = kTN_{\mathrm{s}}\phi_{\mathrm{p,v}}\chi \tag{4}$$

where χ is the polymer–solvent interaction parameter. The equation for the Gibbs free energy change of mixing is

$$\Delta G_{\mathrm{M}} = \Delta H_{\mathrm{M}} - T\Delta S_{\mathrm{M}}. \tag{5}$$

By combining Supplementary equation (5) with Supplementary equations (3) and (4), we can der ive

$$\Delta G_{\mathrm{M}} = kT(N_{\mathrm{p}} \ln \phi_{\mathrm{p,v}} + N_{\mathrm{s}} \ln \phi_{\mathrm{p,s}} + \chi N_{\mathrm{s}}\phi_{\mathrm{p,v}}). \tag{6}$$

The first two terms are always negative, meaning that the mixing is preferred from the aspect of entropy. Therefore, the interaction parameter χ should be small for the homogenous mixing between polymer and solvent, which could be a criterion to choose the proper solvent for the given polymer in a nonpolar system.

Meanwhile, in relation to the solubility parameter, the change of enthalpy during mixing can be also written as:

$$\Delta H_{\mathrm{M}} = V_{\mathrm{s}}\phi_{\mathrm{p,v}}\phi_{\mathrm{s,v}}(\delta_{\mathrm{p}} - \delta_{\mathrm{s}})^2 \tag{7}$$

where V_s is the volume of solvent, δ_p is the solubility parameter of polymer, and δ_p is the solubility parameter of solvent[40]. Combining Supplementary equations (4) and (7) finally gives Equation (1) in main text:

$$\chi = \frac{V_{\mathrm{m,s}}}{RT}(\delta_p - \delta_s)^2 \tag{8}$$

where $V_{m,s}$ is the molar volume of the solvent.

Therefore, the difference of solubility parameters between polymers δ_p and solvents δ_s have to be small for their preferred mixing. The size of solvents is also a variable of mixing preference, because their diffusivity into polymers affects mixing dynamics. Since χ has to be small for preferred mixing, we use the reciprocal value χ^{-1} as a parameter, named the interaction affinity between polymer and solvent. Fig. 2a of the main text and Supplementary Fig. 3 show the χ^{-1} values for the PS and PMMA with various nonpolar solvents, respectively.

3. Relationship between interaction parameter and swelling. When solvent is infiltrated into polymer network, the driving force of swelling is the osmotic pressure π of the polymer solution[22]. First, the free energy of mixing ΔF_M is

$$\Delta F_{\mathrm{M}} = kT(N_{\mathrm{p}} \ln \phi_{\mathrm{p,v}} + N_{\mathrm{s}} \ln \phi_{\mathrm{p,s}} + \chi N_{\mathrm{s}} \phi_{\mathrm{p,v}}). \quad (9)$$

The difference of chemical potential between the pure solvent μ_s^o and the solution μ_s is derived by differentiating equation (9):

$$\Delta\mu = \mu_{\mathrm{s}} - \mu_{\mathrm{s}}^{o} = RT(\ln(1 - \phi_{\mathrm{p,v}}) + (1 - \frac{1}{x})\phi_{\mathrm{p,v}} + \chi\phi_{\mathrm{p,v}}^{2}) \quad (10)$$

where *x* is the number of polymer segment. From the difference of the chemical potential, we can set down the activity of the solvent a_s:

$$a_{\mathrm{s}} = \frac{\Delta\mu}{RT} = (\ln(1 - \phi_{\mathrm{p,v}}) + (1 - \frac{1}{x})\phi_{\mathrm{p,v}} + \chi\phi_{\mathrm{p,v}}^{2}). \quad (11)$$

Since the pure solvent is chosen as the standard state, the activity is the ratio of vapor pressure between the pure solvent P_s and the solution P_s^o, $a_s = P_s/P_s^o$. From the expression of osmotic pressure $\pi = -\Delta\mu/V_{m,s}$, we derive

$$\pi = -\frac{RT}{V_{\mathrm{m,s}}}(\ln(1 - \phi_{\mathrm{p,v}}) + (1 - \frac{1}{x})\phi_{\mathrm{p,v}} + \chi\phi_{\mathrm{p,v}}^{2}). \quad (12)$$

The first two terms on the right are related with the composition of polymer and solvent, while the last term on the right is related with the composition and the component. Thus, the combination of polymer and solvent that have low interaction parameter value can have more high swelling ratio, owing to their higher osmotic pressure.

Fully deformable and soft pure green light-emitting devices

4. The V-PNC@AINO as an efficient color conversion layer. The ideal stretchable perovskite color conversion layer must combine high PLQE and optical density (absorbance) for efficient color conversion, and high stretchability with mechanical softness for low-constrained deformability. We compare the V-PNC@AINO with a nanocomposite consisting of the V-PNC and poly(dimethyl siloxane) (PDMS), which is commercially available siloxane based

PDMS (V-PNC@PDMS), when the concentration of PNC is over 4 mg ml^{-1}, the precursor mixture becomes yellowish, which means an aggregation occurred. Even for lower concentration (~1 mg ml^{-1}), the PLQE of the V-PNC@PDMS is relatively low (~40 %) compared to the V-PNC@AINO with high concentration (~12 mg ml^{-1}) (Supplementary Fig. 37). Also, the V-PNC@PDMSs are environmentally unstable, which lose their luminescence properties ~6 days in air, water, and ~12 hours under UV light. Most importantly, the color coordinate of the device based on V-PNC@PDMS as a color conversion layer is (0.197, 0.390), which has relatively low color purity than that based on the V-PNC@AINO, due to its low optical density and PLQE (Supplementary Fig. 28).

Meanwhile, the V-PNC@AINO efficiently converts blue light to green in the device. As shown in Fig. 4b and Supplementary Fig. 27, the water soaking affects the optical behavior of V-PNC@AINO, where both of the absorbance and PLQE are increased. Therefore, when we use the V-PNC@AINO after 7 days in water as a color conversion layer, the color coordinate of the device is changed from (0.210, 0.625) to (0.192, 0.752), which coordinate is comparable to that of QD-LEDs and Pe-LEDs.

5. Color conversion efficiency of the device. We calculate the color conversion efficiency of the device by dividing the luminance with color conversion layer into the luminance without color conversion layer. As shown in Supplementary Fig. 32, the efficiency of the devices is about 97 % regardless of the applied electric field, due to the superior optical properties of the water-induced PL enhanced V-PNC@AINO.

6. Power conversion efficiency of the device. Since the device emits light from electric signal, we measured the luminous power and the consumed power of the whole device system. First, the luminous power of the device at 3 V μm^{-1} and 4 kHz is calculated to 8.69x10^{-2} lm, which value is obtained by multiplying the measured luminance (34.3 cd m^{-2}) with the measured emission area (7.85x10^{-5} m^{2}), and dividing it into the steradian (0.031 sr) that is calculated from the measurement setup. Then, we calculate the consuming power of the device by measuring the current and the phase difference with the applied voltage. We observe the current by connecting the device with the oscilloscope (DPO5104B, Tektronix) and the low-noise current preamplifier (Stanford research systems, SR570) in series (Supplementary Fig. 34a). As shown in Supplementary Fig. 34b, when the applied root-mean-square voltage V_{RMS} is 2.12 kV, the root-mean-square current I_{RMS} is 0.53 mA. The phase difference is 79.2^{o}, which value is calculated by dividing the time difference at the peak position of the voltage and the current (5.5x10^{-5} s) into the period (2.5x10^{-4} s), and then multiplying 360°. Since the electric power P is equal to $V_{\mathrm{RMS}}I_{\mathrm{RMS}}cos\phi$, the consumption power of the device is 0.21 W. Finally, the luminous efficacy is obtained to 413.6 mlm W^{-1}, by dividing the luminous power into the consumption power. The obtained luminous power value is somewhat higher than previous study[4], possibly due to the existence of $BaTiO_3$ and larger amount of ZnS:Cu phosphors in the ACEL layer.

Supplementary References

33. Hu, H., Salim, T., Chen, B. & Lam, Y. M. Molecularly engineered organic-inorganic hybrid perovskite with multiple quantum well structure for multicolored light-emitting diodes. *Sci. Rep.* **6**, 1-8 (2016).
34. Wei, Z. *et al*. Solution-processed highly bright and durable cesium lead halide perovskite light-emitting diodes. *Nanoscale* **8**, 18021-18026 (2016).
35. Kumar, S. *et al*. Efficient blue electroluminescence using quantum-confined two-dimensional perovskites. *ACS Nano* **10**, 9720-9729 (2016).
36. Kumar, S. *et al*. Ultrapure green light-emitting diodes using two-dimensional formamidinium perovskites: Achieving recommendation 2020 color coordinates. *Nano Lett.* **17**, 5277-5284 (2017).
37. Zhao, F., Kang, Y., Jiang, X. & Peng, X. [Invited paper: quantum dots for display: from photoluminescence to electroluminescence.] in *SID S. Dig. Tech. Pap.*, 2016, Vol. 47, No. 1, pp. 657-659.
38. Yang, Y. *et al*. High-efficiency light-emitting devices based on quantum dots with tailored nanostructures. *Nat. Photonics* **9**, 259-266 (2015).
39. Owens, J. W., Stanley, P. W. & Howard, D. Aqueous solubilities and enthalpies of solution of n-alkylbenzenes. *J. Chem. Eng. Data* **31**, 47-51 (1986).
40. Prak, D. J. L., Prak, P. J. L., Cowart, J. S. & Trulove, P. C. Densities and viscosities at 293.15–373.15 K, speeds of sound and bulk moduli at 293.15–333.15 K, surface tensions, and flash points of binary mixtures of n-hexadecane and alkylbenzenes at 0.1 MPa. *J. Chem. Eng. Data* **62**, 1673-1688 (2017).
41. Prak, D. J. L. Density, viscosity, speed of sound, bulk modulus, surface tension, and flash point of binary mixtures of butylcyclohexane with toluene or n-hexadecane. *J. Chem. Eng. Data* **61**, 3595-3606 (2016).
42. Hansen, C. M. *Hansen solubility parameters: a user's handbook.* (CRC press, 2002).
43. Young, R. J. & Lovell, P. A. *Introduction to polymers.* (CRC press, 2011).
