# Supplementary material for: Aromatic nonpolar organogels for efficient and stable perovskite green emitters
Source: Nat Commun. 2020 Sep 15;11:4638. doi: 10.1038/s41467-020-18383-y (PMC7493929; doi:10.1038/s41467-020-18383-y)
Supplement: Supplementary file 3 — Description of Additional Supplementary Files [file 41467_2020_18383_MOESM3_ESM.pdf]

Description of Additional Supplementary Files

- Supplementary Movie 1: The AINO as transparent, stretchable, and hydrophobic soft matrices.
- Supplementary Movie 2: Tensile tests of the AINO and V-PNC@AINO.
- Supplementary Movie 3: Stretching the V-PNC@AINO after 70 days in water.
- Supplementary Movie 4: Stretch-and-release cyclic test of the PNC@AINOs.
- Supplementary Movie 5: Demonstration of fully deformable pure green light-emitting device.
